# Configuration-Specific Temporal Alignment of a Repurposed Wearable Fuel-Cell Alcohol Sensor with an MQ-3 Timing Proxy in a Laboratory Static-Headspace Chamber

**DOI:** 10.3390/s26216949

**Published:** 2026-11-03

**Authors:** Hasan Mirzakhaninafchi, Glen Rains, Wesley Porter, Canicius Mwitta, Denis Kiobia, Shekhar Thapa, Peter Ngimbwa, Christopher Ranger

**Affiliations:** 1 Department of Entomology, University of Georgia, Tifton, GA 31793, USA; grains@uga.edu (G.R.); cmwitta@uga.edu (C.M.); denis.kiobia@uga.edu (D.K.); 2 Department of Crop and Soil Sciences, University of Georgia, Tifton, GA 31793, USA; wporter@uga.edu; 3 College of Engineering, University of Georgia, Tifton, GA 31793, USA; shekhar.thapa@uga.edu (S.T.); peter.ngimbwa@uga.edu (P.N.); 4 Application Technology Research Unit, United States Department of Agriculture, Agricultural Research Service, 1680 Madison Avenue, Wooster, OH 44691, USA; christopher.ranger@usda.gov

**Keywords:** ethanol sensing, BACtrack Skyn, MQ-3, wearable fuel-cell sensor, metal-oxide sensor, static headspace, temporal alignment, denatured alcohol, tree-monitoring development

## Abstract

Ethanol emitted from stressed woody tissues is a host-associated cue for ambrosia beetles, motivating investigation of portable ethanol-responsive sensors for future tree monitoring. This laboratory study evaluated temporal alignment between two asynchronously logged volatile organic compound (VOC)-responsive sensors during a denatured-alcohol chamber challenge. We selected the BACtrack Skyn wearable fuel-cell alcohol sensor for its compact form and timestamped output as a candidate for future tree-headspace measurements, and evaluated one device with an MQ-3 metal-oxide timing proxy and environmental logging in a closed static-headspace chamber. The dataset comprised five alcohol-free control runs (B0–B4) and three technical 1 μL denatured-alcohol challenge runs (EtOH1–EtOH3). Signals were processed on a common 1 min grid using a 10–15 min analytical baseline and a 15–45 min response-characterization window. MQ-3 peaks occurred at 17.0–17.3 min from the run origin ($T_{0}$), whereas Skyn device-output peaks occurred at 18.8–19.2 min; peak-time offsets were 1.5–2.2 min. First-difference analysis selected a 2 min alignment setting on the primary 1 min grid in all three challenge runs; a supplementary 20 s analysis gave run-specific optima of 80–160 s. Across the three challenge runs, run-specific Pearson correlations ranged from 0.686 to 0.751 before alignment and from 0.861 to 0.930 after alignment; each coefficient was calculated from the paired 1 min observations in the 15–45 min response window. Normalized root-mean-square error (RMSE) ranged from 0.208 to 0.272 before alignment and from 0.121 to 0.230 after alignment, whereas rank correlation did not improve consistently. Alcohol-free controls remained below challenge-run peak magnitudes, although MQ-3 traces included drift and positive excursions. These within-run comparisons describe alignment in the tested configuration, not intrinsic sensor latency, concentration calibration, or validated tree-monitoring performance.

## 1. Introduction

Ethanol is a stress-induced volatile associated with altered metabolism in woody tissues in response to abiotic and biotic factors, including flooding, extreme temperatures, and pathogen infection. In plants, ethanol can be produced when aerobic metabolism is impaired and fermentative pathways are induced, and its accumulation has been documented as an early indicator of hidden tissue injury in woody systems [1,2]. In parallel, ethanol is a biologically important host-associated cue in ambrosia beetle systems, where stressed trees growing in horticultural production systems can become more attractive and susceptible to colonization [3,4,5].

Ethanol can also influence post-selection outcomes in fungus-farming ambrosia beetle systems. Ethanol-rich substrates can favor nutritional fungal symbionts and suppress competing fungi, thereby affecting colonization outcomes and gallery ecology. Thus, ethanol can function both as an attraction cue and as an indicator of host condition and colonization context [6,7]. Recent experiments also found that ethanol tolerance influenced the efficacy of fungal biocontrol strains against ambrosia beetles [8]. Recent work on flood-stressed flowering dogwood compared low-cost alcohol detectors with solid-phase microextraction–gas chromatography–mass spectrometry (SPME–GC–MS) and found positive associations with analytically confirmed ethanol, while also documenting false-positive alcohol-detector responses associated with plant volatiles [9]. This evidence supports the motivation for low-cost sensing but also shows that chemical selectivity and field validity cannot be inferred from a denatured-alcohol chamber challenge. Commercial indoor-air-quality sensors have also been evaluated using individual terpenes and emissions from living basil plants [10].

Wearable fuel-cell alcohol sensors provide a compact alternative for ethanol-responsive time-series sensing. BACtrack Skyn was selected for its compact form and timestamped alcohol-output records, which could support repeated measurements in tree-headspace enclosures [11,12]. Its suitability for tree monitoring was not assumed from its original transdermal application. Human transdermal measurements include ethanol transport through tissue and skin before detection; this transport pathway is absent during direct headspace exposure [11]. However, their outputs require careful preprocessing and temporal interpretation because they are delayed and smoothed relative to faster alcohol measurements and can contain artifacts, missing segments, and environmental contamination [11,12,13]. A repurposed wearable alcohol sensor may, therefore, be useful for tree-relevant headspace monitoring only if its timing behavior is characterized in the deployment configuration of interest.

Despite strong biological motivation for ethanol monitoring, time-resolved ethanol measurement at tree scale remains difficult. Conventional volatile measurements often depend on laboratory-centered instrumentation or dynamic chamber systems that require careful material selection, stable flow control, and environmental monitoring. Such systems can generate high-quality data, but they are not always practical for repeated, low-burden measurements across many individual trees or headspace configurations [14,15].

This laboratory study was motivated by the need to compare asynchronous alcohol-responsive sensor streams before integrating repurposed wearable sensing into tree-monitoring experiments. The key methodological question was whether the two channels showed comparable response timing under repeated controlled challenges after applying one configuration-specific alignment shift. The study, therefore, focused on cross-sensor timing mismatch, peak timing, first-difference alignment, response-shape comparison, and alcohol-free control context. It did not address ppm calibration, VOC selectivity, direct measurement of internal woody-tissue ethanol, or prediction of ambrosia beetle attack.

Specifically, the objectives were to (i) compare the responses of a repurposed BACtrack Skyn and an MQ-3 timing proxy during three controlled 1 μL denatured-alcohol challenges and five alcohol-free control runs; (ii) quantify run-specific peak-time and response-shape offsets; (iii) evaluate one shared alignment shift across the three challenge runs and (iv) assess alignment using correlation, normalized shape error, peak timing, and onset-offset sensitivity diagnostics. The methodological contribution is an application-specific assessment of temporal alignment for a repurposed wearable sensor and an independently logged metal-oxide channel. Common-time-base processing is combined with separate peak-time, response-shape, and threshold-onset comparisons and with temporal-grid sensitivity analysis. In these records, a shared 2 min shift improved peak and response-shape agreement on the primary 1 min grid, but did not provide a uniform threshold-onset correction; finer-grid optima also differed among runs. These findings delimit the interpretation of the alignment setting for this configuration. The study does not introduce a new sensing material, transducer design, or lag-estimation algorithm. It evaluates the temporal comparability of the recorded outputs, not concentration accuracy, chemical selectivity, or a biological decision threshold.

## 2. Materials and Methods

### 2.1. Experimental Overview

This study was a laboratory measurement-alignment experiment using a fixed static-headspace configuration containing one BACtrack Skyn wearable fuel-cell alcohol sensor (BACtrack, San Francisco, CA, USA), one MQ-3 metal-oxide sensor module (HUABAN; supplier: Shenzhen Eric Electronics Technology Limited, Shenzhen, China), one BME280 environmental sensor (Bosch Sensortec GmbH, Reutlingen, Germany), and one Type-K thermocouple probe connected through a MAX6675 interface module (DEVMO; supplier: developmoreus, Shenzhen, China). The MQ-3 and environmental channels were recorded using an ESP32 microcontroller (Espressif Systems, Shanghai, China). The dataset comprised five alcohol-free control runs (B0–B4) and three technical 1 μL denatured-alcohol challenge runs (EtOH1–EtOH3). All runs were analyzed using the same baseline and response-window definitions where data coverage allowed. No alcohol was added during the control runs. B0 and B2 did not provide complete Skyn coverage through 45 min and were interpreted accordingly in response-magnitude and quality-control summaries.

The Skyn was treated as the wearable ethanol-responsive fuel-cell channel. The MQ-3 was used as an uncalibrated timing proxy for within-configuration response comparison; it was not treated as an ethanol-selective analytical reference or concentration standard. Metal-oxide responses can be affected by humidity, temperature, sensor drift, heater state, and chamber history [16]. The analytical target was temporal comparability and response-shape similarity, not concentration equivalence. Environmental measurements from the BME280 and thermocouple channel were retained as confound checks and were not used to fit an environmental correction model.

### 2.2. Headspace Enclosure and Chamber Geometry

A custom headspace enclosure was used to provide repeatable local exposure geometry during controlled ethanol challenges and potential tree-adjacent deployments. The enclosure function in this study was not to estimate flux, but to standardize the airspace seen by the sensors so that cross-sensor timing mismatch could be measured under controlled conditions. Here, flux denotes an emission rate per unit source area, whereas headspace concentration denotes the amount of analyte per unit gas volume. Neither quantity was determined; the measured variables were MQ-3 ADC response and Skyn device output. This logic follows the broader enclosure literature, which emphasizes that material choice, geometric consistency, and, for dynamic chamber systems, flow stability are prerequisites for interpretable VOC measurements [14,15].

The standardized headspace enclosure was fabricated by fused-deposition 3D printing from gray polylactic acid (PLA). The internal geometry included a central cuboid chamber measuring 3.3×7.2×14.9 cm and a cylindrical stem-through channel with an overall internal end-to-end length of 30.5 cm and an internal diameter of 2.7 cm. The enclosure had a nominal wall thickness of 2.5 mm and was assembled using matched 3D-printed frame interfaces with an elastic silicone sealing layer at the mating surfaces and cylindrical side joints to reduce leakage risk and maintain repeatable closure during controlled ethanol challenges. Because the enclosure was used for timing alignment rather than flux estimation, sealing was treated as a practical means of achieving repeatable closure rather than as a basis for absolute concentration or emission-rate claims.

The nominal unoccupied geometric volume was approximately 0.44 L, calculated by adding the cylindrical extensions outside the 14.9 cm central chamber to the cuboid volume. This value does not account for displacement by installed components, does not represent the measured operating air volume, and was not used to infer gas-phase concentration. No tree, woody tissue, or physical stem surrogate occupied the channel during the recorded runs. The channel ends were closed.

**Figure 1:**
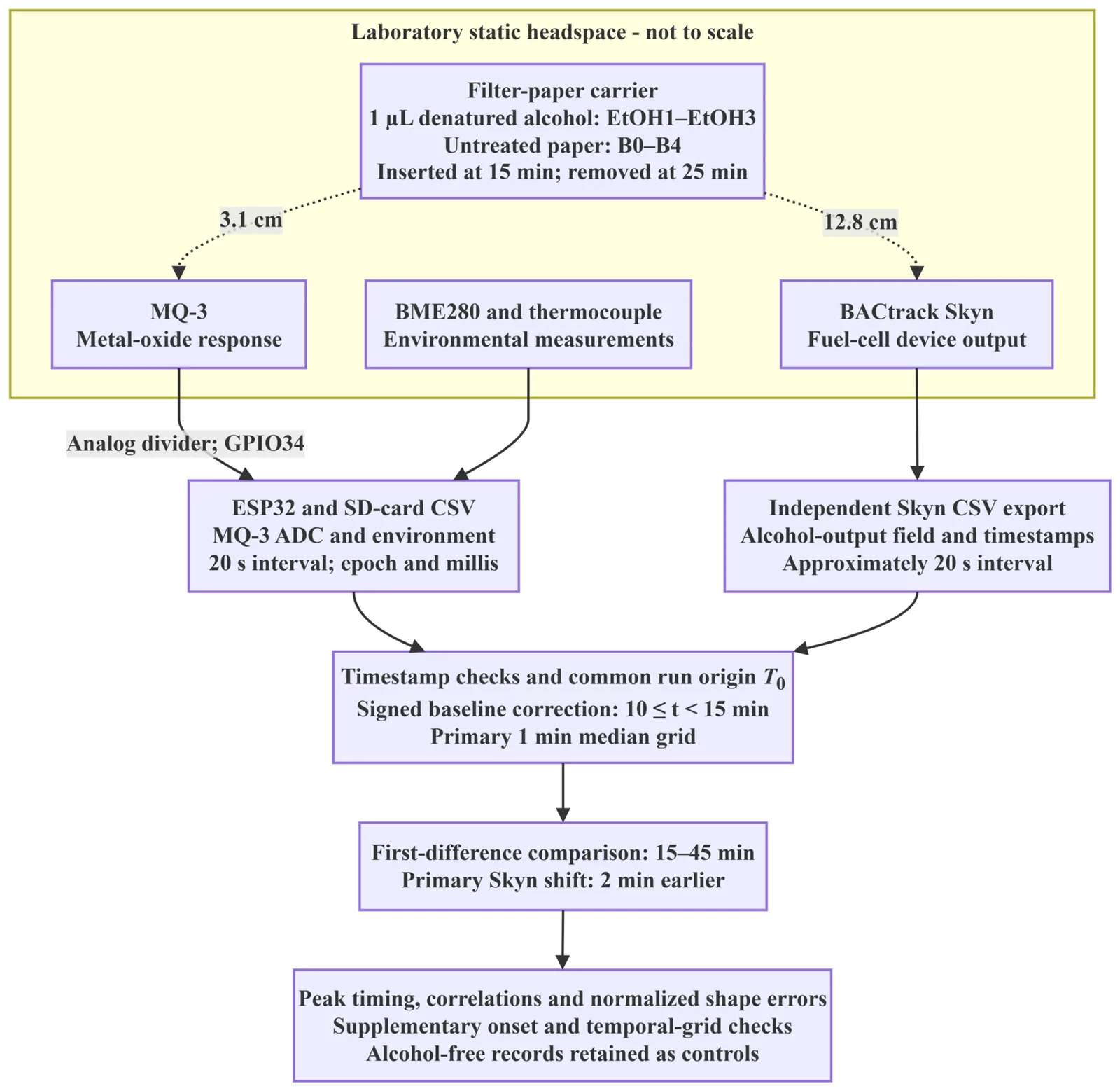
Laboratory static-headspace components and acquisition/alignment workflow. The diagram is functional and not to scale. Dashed connections indicate the source-to-sensor relationships and the measured distances in Section 2.4; they do not imply identical vapor-arrival times. Solid arrows indicate acquisition and processing pathways. The stem-through channel was empty during the recorded runs. MQ-3 and environmental measurements were logged by the ESP32; Skyn records were exported independently. The primary analysis used a common 1 min grid and a two-bin alignment setting. Neither output was interpreted as independently calibrated chamber-air concentration.

The laboratory configuration used a closed, static headspace interface without active flow control. Therefore, the enclosure was interpreted as a repeatable exposure geometry for timing comparison rather than as a dynamic chamber for flux estimation or calibrated headspace concentration measurement. The laboratory configuration is summarized in Figure 1 and Table 1. Annotated interior and exterior views of the apparatus are provided in Figure S7.

### 2.3. Sensor Hardware and Placement

The Skyn uses an electrochemical fuel cell in which alcohol oxidation produces an electrical current [11,17]. The MQ-3 uses a heated tin dioxide (SnO_2_) sensing layer whose electrical resistance changes during exposure to alcohol vapor [18]. The sensitive elements are, therefore, an electrode–electrolyte system in Skyn and a heated semiconducting oxide in MQ-3. This study analyzed the exported Skyn alcohol field and the MQ-3 module output recorded as ADC counts; fuel-cell current and sensing-layer resistance were not measured directly.

The ESP32 logging stack included a BME280 environmental sensor, a MAX6675 thermocouple interface, and an analog MQ-3 input. The firmware wrote run identifiers, epoch seconds when network time synchronization succeeded, a monotonic millisecond counter, BME280 temperature, relative humidity, pressure, thermocouple temperature, MQ-3 raw ADC, MQ-3 ADC-voltage proxy, startup MQ-3 baseline values, and baseline-referenced MQ-3 relative response to the SD card at a 20 s interval. In the analyzed files, NTP-derived epoch timestamps were valid for all retained MQ-3 rows, and the median ESP32 sampling interval was 20.00 s for B0–B4 and EtOH1–EtOH3.

The MQ-3 module was powered from 5 V with common ground to the ESP32 logger. Its analog output was attenuated with a 22 kΩ/33 kΩ voltage-divider interface (AO–22 kΩ–GPIO34–33 kΩ–ground) and read on ESP32 ADC1 GPIO34 as 12-bit analogRead counts from 0 to 4095. After logger and sensor initialization, the firmware applied a fixed 600 s MQ-3 warm-up. It then calculated the startup baseline as the arithmetic mean of 200 analog samples collected at nominal 50 ms intervals, and subsequent MQ-3 response values were reported relative to this baseline. No signal-stability criterion was used to terminate the warm-up or accept the startup baseline. The fixed warm-up was a procedural setting and was not interpreted as evidence of full sensor stabilization. The millis_ms counter began anew for each retained recording, consistent with a separate ESP32 boot or reset; this does not establish whether MQ-3 heater power was interrupted. The available acquisition files and analysis metadata did not document longer-term MQ-3 burn-in, chamber cleaning or ventilation between recordings, a signal-based recovery or washout criterion, or randomization. The MQ-3 module was treated as a low-cost ethanol-responsive timing channel and not as a calibrated concentration sensor [18]. No gas-specific calibration curve, humidity-correction model, or ADC-to-ppm conversion was applied.

The Skyn export files contained timestamped device-reported alcohol values. All analyzed Skyn files came from a single device with firmware version 4.13.1 and app version 1.0.34+2. The exported alcohol field was labeled tac (μg/L); this label is retained as a device-output field name and is not interpreted as an independently calibrated chamber-air concentration. The median Skyn export interval was 20 s, with observed intervals of 19–21 s in the analyzed raw exports. Raw Skyn exports also contained account- and device-identifying fields; these fields were retained only in restricted provenance records and were excluded from analysis tables intended for public release. Skyn records were sorted chronologically, parsed in the America/New_York time zone, and aligned to the MQ-3 $T_{0}$ defined as the first retained ESP32/MQ-3 logging sample after the MQ-3 settling period and startup baseline calibration. The Skyn and MQ-3 were mounted in fixed positions within the same enclosure so that both channels recorded the same scheduled chamber challenge.

### 2.4. Ethanol Challenge Design

Each run was analyzed relative to $T_{0}$, the first retained MQ-3 sample after the MQ-3 settling period and startup baseline calibration. The initial quality-control interval was 0–15 min, the local analytical baseline was 10–15 min, the challenge interval was 15–25 min, and the response-characterization window was 15–45 min. For EtOH1–EtOH3, the challenge interval corresponded to a 1 μL denatured-alcohol challenge. For B0–B4, it was the corresponding alcohol-free analysis interval. For local baseline correction and response-magnitude summaries, the 10–15 min analytical baseline was implemented as the half-open interval 10≤t<15 min.

A single nominal dose was used to hold source loading constant while testing whether the temporal offset was repeatable across technical runs. The 1 μL denatured-alcohol challenge was treated as a repeatable perturbation rather than as a vapor-concentration standard; the study did not test whether the offset was independent of ethanol concentration. EtOH1–EtOH3 were technical repetitions at the same nominal liquid loading, not samples prepared at three ethanol concentrations. In each run, 1 μL of denatured ethyl alcohol (Innovating Science IS14018, labeled 95%, lab grade; Aldon Corporation, Avon, NY, USA) was dispensed with a 0.5–10 μL adjustable micropipette (ONiLAB, Hangzhou Onilab Laboratory Instrument Co., Hangzhou, Zhejiang, China) onto one 46 mm StonyLab quantitative medium circular filter-paper disc (StonyLab Inc., Nesconset, NY, USA). The product safety data sheet lists ethanol (85.9%), water (5.0%), isopropyl alcohol (4.8%), and methanol (4.3%) [19]. The supplier’s specification sheet differs in denaturant composition [20]; the SDS percentages are not treated as a verified batch analysis or chamber-air composition. Contributions from denaturants cannot be excluded, so the responses cannot be assigned exclusively to ethanol. The treated carrier was introduced at 15 min from $T_{0}$ and placed in the same nominal source position in each challenge run. The measured straight-line distance from the carrier center to the exposed MQ-3 sensing element was 3.1 cm, and the distance to the Skyn sensing surface was 12.8 cm. The carrier and the MQ-3, Skyn, BME280, and thermocouple sensing positions remained fixed across the challenge runs. During insertion, the enclosure was opened briefly, the carrier was inserted, and the enclosure was resealed. The carrier remained in the enclosure during the 15–25 min challenge interval and was removed at 25 min. The subsequent 25–45 min interval was a post-removal observation period, not a controlled zero-ethanol exposure. No alcohol was added during B0–B4. For these controls, the enclosure was opened at 15 min, an untreated 46 mm filter-paper disc was placed at the same source position, and the enclosure was resealed. At 25 min, the enclosure was reopened, the disc was removed, and the enclosure was resealed. The same analytical time windows were used for controls and challenges. Because denatured ethyl alcohol was used, the challenge was interpreted as a mixed alcohol/VOC perturbation, not chemically pure ethanol vapor. Vapor was generated by evaporation from the treated filter-paper carrier; no vial or effusion cell was used. Chamber temperature was monitored as environmental context (Table S4), but these measurements do not establish the liquid-source temperature or evaporation rate. No independent vapor-concentration standard, dynamic-flow measurement, or ppm calibration was used. Thus, the protocol specified nominal liquid loading and source position without defining a calibrated vapor-concentration profile. The recording order and inter-recording gaps are reported in Table S6.

### 2.5. Data Acquisition and Timestamp Handling

MQ-3 and environmental data were acquired through the ESP32 logger at a 20 s interval. The firmware created both absolute and monotonic timing fields: epoch seconds when NTP synchronization succeeded and a monotonic millisecond counter regardless of network time. For processing, the epoch timestamp was preferred when valid, and millis_ms was retained as an independent monotonic timing reference. In all retained ESP32/MQ-3 files analyzed here, the epoch-valid fraction and NTP-valid fraction were both 1.0.

Skyn timestamps were parsed from exported device records, converted into the analysis time zone, sorted chronologically, and restricted to the corresponding MQ-3 time window plus padding. MQ-3 and Skyn traces were resampled to a common 1 min time grid using median aggregation. The 1 min grid was used for lag estimation, paired response-window statistics, normalized response-shape comparison, and plotting. Preprocessing and alignment parameters were recorded for each run. Original raw filenames and firmware run identifiers were retained in the analysis records; canonical labels B0–B4 and EtOH1–EtOH3 were assigned for manuscript reporting. Recorded MQ-3 ADC values for EtOH2 are shown in Figure S6; the corresponding acquisition record and processed paired 1 min series are provided as Data S1.

### 2.6. Preprocessing and Quality Control

MQ-3 preprocessing began with a signed relative response computed from raw ADC and the firmware-recorded startup baseline:


(1) $$R_{\mathrm{MQ}3}\left(t\right)=\frac{{\mathrm{ADC}}_{\mathrm{raw}}\left(t\right)-{\mathrm{ADC}}_{\mathrm{baseline},\mathrm{startup}}}{{\mathrm{ADC}}_{\mathrm{baseline},\mathrm{startup}}}.$$

Here, *t* denotes elapsed time from $T_{0}$, ${\mathrm{ADC}}_{\mathrm{raw}}\left(t\right)$ is the raw 12-bit MQ-3 ADC reading at time *t*, ${\mathrm{ADC}}_{\mathrm{baseline},\mathrm{startup}}$ is the run-level startup baseline computed as the arithmetic mean of 200 ADC samples, and $R_{\mathrm{MQ}3}\left(t\right)$ is the dimensionless signed relative MQ-3 response.

The analytical MQ-3 timing signal was then locally baseline-zeroed using the median response during the 10–15 min pre-exposure analytical baseline:


(2) $${\mathrm{MQ}3}_{\mathrm{sig}}\left(t\right)=R_{\mathrm{MQ}3}\left(t\right)-\mathrm{median}\left\{R_{\mathrm{MQ}3}\left(u\right):10\leq u<15\right\}.$$

Here, $\mathrm{MQ}3_{\mathrm{sig}}\left(t\right)$ is the signed local-baseline-corrected MQ-3 response, and *u* is a dummy elapsed-time variable indexing observations in the half-open analytical baseline interval 10≤u<15 min.

This signed, local-baseline-corrected signal was used as the MQ-3 timing proxy. Negative excursions were retained rather than clipped because baseline drift and recovery behavior were relevant quality-control information. A centered rolling median with a 5-sample window was used for visualization only. For response-magnitude summaries and the associated figures, raw ADC values were also expressed as local-baseline-subtracted differences:

(3) $$\Delta {\mathrm{ADC}}_{\mathrm{MQ}3}\left(t\right)={\mathrm{ADC}}_{\mathrm{raw}}\left(t\right)-\mathrm{median}\left\{{\mathrm{ADC}}_{\mathrm{raw}}\left(u\right):10\leq u<15\right\}.$$

Here, $\Delta {\mathrm{ADC}}_{\mathrm{MQ}3}\left(t\right)$ is the local-baseline-subtracted MQ-3 response in ADC counts; *u* has the same meaning and baseline interval defined above.

Two MQ-3 representations were used for distinct purposes. The signed relative MQ-3 response was used for temporal-alignment, correlation, and normalized response-shape analyses. Local-baseline-subtracted raw ADC differences were reported separately for response-magnitude summaries and figures. The two representations were not combined within a single statistical calculation.

Skyn TAC values were processed using predefined quality-control rules. TAC values were coerced to numeric values, and predefined validity filters excluded non-positive values and values at or above 500 μg L^−1^ in device-export units before resampling. Records were required to include at least 30 retained points and at least 10 unique TAC values after cleaning. A dominant repeated TAC value would be excluded if it represented more than 50% of retained rows. For relative response-shape analysis, Skyn TAC was baseline referenced to the median TAC during the same 10–15 min analytical baseline when sufficient baseline points were available:


(4) $${\mathrm{Skyn}}_{\mathrm{rel}}\left(t\right)=\frac{\mathrm{TAC}(t)-\mathrm{median}\{\mathrm{TAC}(u):10\leq u<15\}}{\mathrm{median}\{\mathrm{TAC}(u):10\leq u<15\}}.$$

Here, TAC(t) denotes the vendor-exported Skyn alcohol-output field at elapsed time *t*, ${\mathrm{Skyn}}_{\mathrm{rel}}\left(t\right)$ is the dimensionless local-baseline-normalized Skyn response, and *u* indexes observations in the half-open analytical baseline interval.

For response-magnitude summaries, local-baseline-subtracted Skyn device output was calculated as


(5) $$\Delta S_{\mathrm{Skyn}}\left(t\right)=\mathrm{TAC}\left(t\right)-\mathrm{median}\left\{\mathrm{TAC}(u):10\leq u<15\right\}.$$

Here, $\Delta S_{\mathrm{Skyn}}\left(t\right)$ is the local-baseline-subtracted Skyn response in device-export units.

Signed ${\mathrm{Skyn}}_{\mathrm{rel}}\left(t\right)$ values were used for timing, correlation, and quality-control diagnostics. The baseline-normalized Skyn response was used for temporal-alignment and cross-sensor response-shape analyses, whereas local-baseline-subtracted device output was used for response-magnitude summaries. Skyn output was treated as a device-reported response variable and not as a gas-phase concentration. Preprocessing parameters are summarized in Table 2.

### 2.7. Lag Estimation Methods

Here, alignment offset denotes a numerical shift selected on a specified analysis grid. Peak-time difference denotes the difference between observed peak times, with native-record and resampled-grid values reported separately. Onset offset denotes the difference between the threshold crossings defined in Section 2.10. Vapor transport and sensor/device response are possible contributors to the recorded timing differences, but were not separately measured; intrinsic sensor latency was not estimated.

Lag estimation was performed on the monotonic 1 min analysis time axis rather than relying only on calendar timestamps. For EtOH1–EtOH3, the primary lag diagnostic was a first-difference estimator computed from the 1 min resampled MQ-3 and signed Skyn relative traces within the 15–45 min response-characterization window. The primary search covered 0–10 min in 1 min steps, with positive values indicating that Skyn trailed MQ-3. First differences, $\Delta x_{i}=x_{i}-x_{i-1}$, were calculated on each complete resampled record before selecting grid labels 15 through 45 inclusive from both difference sequences. Here, $x_{i}$ denotes the signed response of either sensor. Candidate offsets were compared by Pearson correlation of at least 20 overlapping finite pairs (Figure S3). The primary 2 min setting, therefore, represents two 1 min bins, not a delay measured to 0.1 min precision. As a directional-sensitivity analysis, the first-difference search was repeated over −10 to +10 min. A cumulative-trace estimator and timestamp-based estimator were retained as supplementary diagnostics but were not used to define the reported shared alignment shift.

For the reported figures and paired statistics, one shared alignment shift was applied across the three challenge runs rather than optimizing a separate shift for each run. The shared alignment shift was defined from the challenge-run first-difference diagnostics and applied as 2 min across EtOH1–EtOH3. Alcohol-free controls were used only to characterize background behavior and were not used to estimate the challenge-response alignment offset.

A retrospective leave-one-run-out check selected the median of the two training-run first-difference optima and applied that shift to the held-out run without re-optimization. All other processing settings were held fixed. Because those settings had been developed using the complete dataset, this was a sensitivity check, not prospective validation.

A supplementary analysis used the native records aggregated into common 20 s median bins anchored to $T_{0}$ and labeled by their lower edges. The baseline definitions were unchanged. Consecutive first differences were calculated before restricting both sequences to 15–45 min. Candidate offsets from −10 to +10 min were evaluated in 20 s steps using at least 60 overlapping finite pairs; missing bins were not filled. Bin-placement sensitivity was assessed by moving the bin origin from $T_{0}$ by 0–19 s in 1 s increments while retaining the same baseline and response windows. The resulting ranges describe numerical sensitivity, not statistical confidence intervals. Changing the grid also changes the interval over which consecutive differences are calculated.

### 2.8. Alignment and Merge Procedure

After offset estimation, Skyn time was shifted earlier by 2 min so that a positive offset represented the amount by which Skyn trailed MQ-3. Shifted Skyn values were paired with MQ-3 values on the common 1 min time grid for response-window statistics. Nearest-neighbor merged files with a 2 min tolerance were exported only for quality control and traceability and were not used for the primary paired statistics.

### 2.9. Paired MQ-3/Skyn Response-Window Analysis

The primary cross-sensor analysis used paired 1 min MQ-3 and Skyn values within predefined analysis windows before and after the shared alignment shift. Pearson correlation, Spearman rank correlation, normalized bias, RMSE, MAE, and within-window peak-time differences were calculated for each challenge run. For the normalized shape errors, let $x_{i}=\mathrm{MQ}3_{\mathrm{sig}}\left(t_{i}\right)$ and $y_{i}={\mathrm{Skyn}}_{\mathrm{rel}}\left(t_{i}+L\right)$, with L=0 before alignment and L=2 min after alignment. Finite paired grid labels 15 through 45 inclusive were used (n=31 in each challenge run). For each alignment condition separately,

(6) $$P_{x}=\max_{i}\left\{\max\left(x_{i},0\right)\right\},\;P_{y}=\max_{i}\left\{\max\left(y_{i},0\right)\right\},\;{\hat{x}}_{i}=\frac{x_{i}}{P_{x}},\;{\hat{y}}_{i}=\frac{y_{i}}{P_{y}},\;d_{i}={\hat{y}}_{i}-{\hat{x}}_{i}.$$

Both maxima were positive in the challenge runs. Signed values were retained after division. The dimensionless error summaries were

(7) $$\mathrm{bias}=\frac{1}{n}\sum_{i=1}^{n}d_{i},\;\mathrm{RMSE}=\sqrt{\frac{1}{n}\sum_{i=1}^{n}d_{i}^{2}},\;\mathrm{MAE}=\frac{1}{n}\sum_{i=1}^{n}\left|d_{i}\right|.$$

Moving-block bootstrap intervals and exact circular-shift calculations were retained as within-run sensitivity diagnostics [21,22]. They were not interpreted as population-level tests because the study used one device pair and three technical challenge runs. These analyses quantify temporal response similarity; they are not raw-unit agreement or concentration-calibration tests.

### 2.10. Window-Level Onset-Offset Summaries

Threshold-based onset analysis was retained as a sensitivity diagnostic rather than as the primary endpoint. After centered 3 min rolling-median smoothing of the timestamp-resampled 1 min series, onset was defined as the first sample at or above threshold within the 15–25 min window for MQ-3, unshifted Skyn, and shifted Skyn. For this supplementary diagnostic, baseline standard deviations were calculated from the smoothed series over the inclusive 10–15 min interval, with windows referenced to the first MQ-3 timestamp bin. MQ-3 and Skyn thresholds were defined as the larger of 3 times the baseline standard deviation or the minimum absolute threshold: 0.05 for MQ-3 and 0.20 for Skyn relative response. Offsets were expressed as Skyn onset minus MQ-3 onset in minutes. Because onset timing is sensitive to threshold choice and 1 min discretization, onset results were interpreted as a supplementary timing check rather than the primary alignment statistic. The centered filter incorporates later observations, and the inclusive baseline can include response values near the nominal insertion boundary. These retrospective threshold crossings, therefore, do not establish physical vapor-arrival times or real-time detection latency.

### 2.11. Statistical Analysis

This study reports descriptive and diagnostic statistics for five alcohol-free control runs and three technical denatured-alcohol challenge runs. The principal challenge-run outputs were MQ-3 and Skyn peak timing, Skyn-minus-MQ-3 peak offset, local-baseline-subtracted response magnitude, first-difference alignment offset, paired response-window Pearson and Spearman correlations, normalized response-shape bias and RMSE, and onset-offset sensitivity metrics. Control-to-challenge comparisons were interpreted descriptively because the dataset contained a small number of technical runs under one static-headspace configuration. No generalized concentration-calibration model was fitted. Analyses were conducted in Python 3.12.13 using pandas 2.2.2, NumPy 2.0.2, and Matplotlib 3.10.0.

Because the shared 2 min shift was estimated from the same challenge runs to which it was applied, the after-shift statistics are interpreted as internal alignment diagnostics rather than as independent validation of a transferable offset.

The bootstrap and circular-shift calculations were used as within-run sensitivity diagnostics and were not interpreted as population-level evidence because the experiment used one device pair and three technical challenge runs.

## 3. Results

### 3.1. Alcohol-Free Control and Denatured-Alcohol Challenge Responses

**Figure 2:**
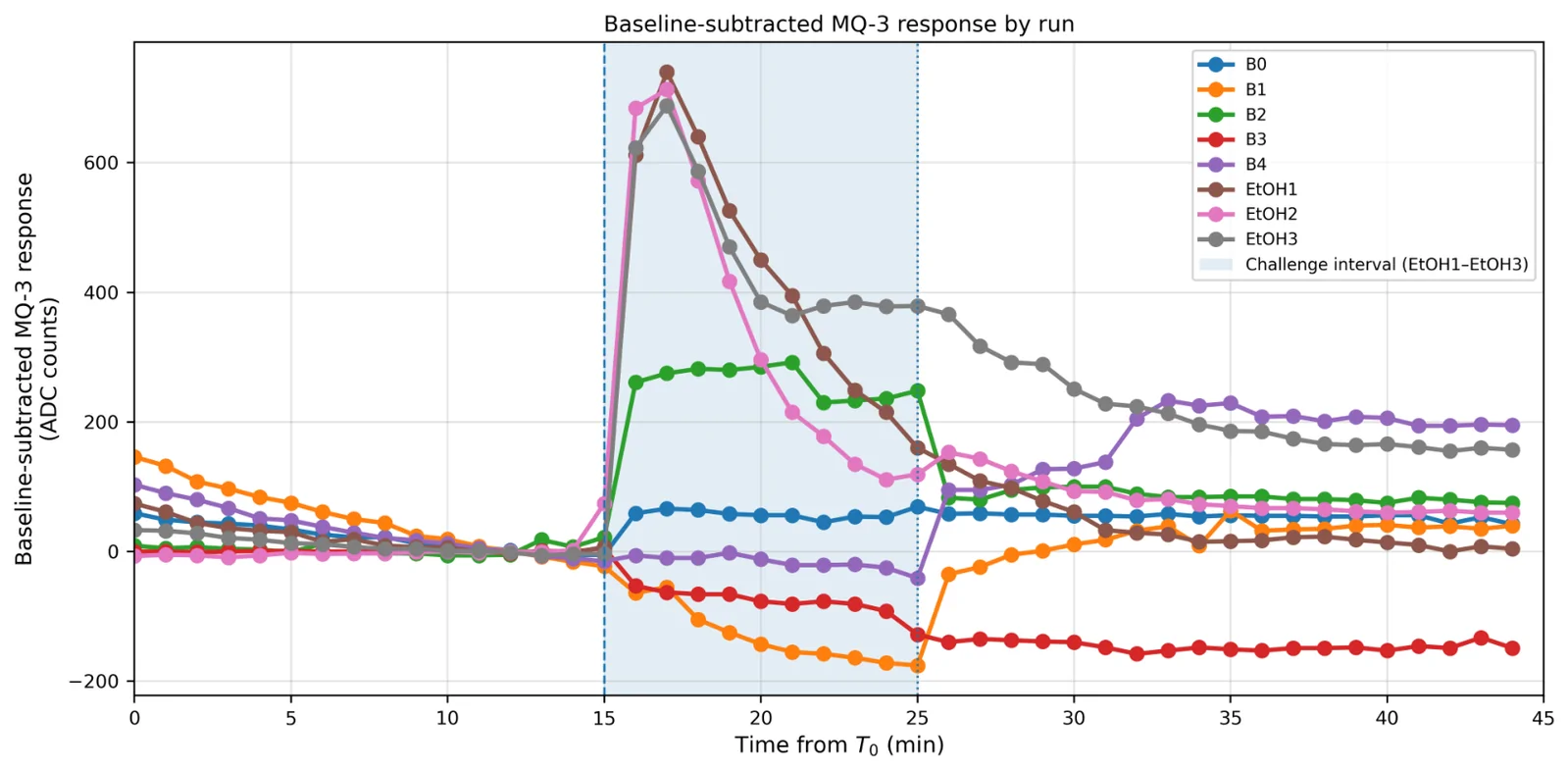
Local-baseline-corrected MQ-3 response traces for alcohol-free runs B0–B4 and technical 1 μL denatured-alcohol challenge runs EtOH1–EtOH3. No alcohol was added during B0–B4. The shaded 15–25 min interval indicates the presence of the alcohol-treated carrier in EtOH1–EtOH3 and the untreated carrier in B0–B4. No temporal shift was applied to the MQ-3 traces. B1 and B3 showed negative MQ-3 drift, whereas B2 and B4 showed positive MQ-3 excursions. Challenge runs showed larger and more temporally clustered positive peak deltas. The MQ-3 was used as an uncalibrated timing proxy rather than as a concentration reference.

Five alcohol-free controls were analyzed to characterize background behavior. Figure 2 shows the local-baseline-corrected MQ-3 traces, and Table 3 summarizes response magnitudes and Skyn coverage for all runs. Skyn positive peak deltas ranged from 0.324 to 0.416 μg L^−1^ export units for B0–B4, compared with 102.426–288.348 μg L^−1^ export units for EtOH1–EtOH3. MQ-3 positive peak deltas ranged from 7 to 308 ADC in the alcohol-free controls and from 718 to 756 ADC in the challenge runs. B1 and B3 showed negative MQ-3 drift, whereas B2 and B4 showed positive MQ-3 excursions without corresponding challenge-scale Skyn responses. All challenge-run MQ-3 and Skyn positive peak deltas exceeded the corresponding alcohol-free ranges. Figure S4 shows that structured MQ-3 variation occurred without alcohol addition. The available Skyn exports for B0 and B2 ended at 43.45 and 39.55 min from $T_{0}$, respectively; their summaries may, therefore, omit later excursions. The cause of this data-completeness limitation was not established.

### 3.2. Environmental Context and Confound Checks

Environmental variables were monitored across all runs as confound context and are summarized in Table S4. During the 15–45 min response-characterization window, BME280 temperature remained within 24.13–25.26 °C across runs and relative humidity remained within 52.798–60.482%. Environmental traces showed gradual run-level variation rather than a consistent event-shaped pattern paralleling the challenge-run sensor responses. Because the dataset was small and not designed for environmental model fitting, environmental variables were used as quality-control and confound checks rather than as correction-model predictors.

### 3.3. Lag Diagnostics and Shared Alignment Offset

**Figure 3:**
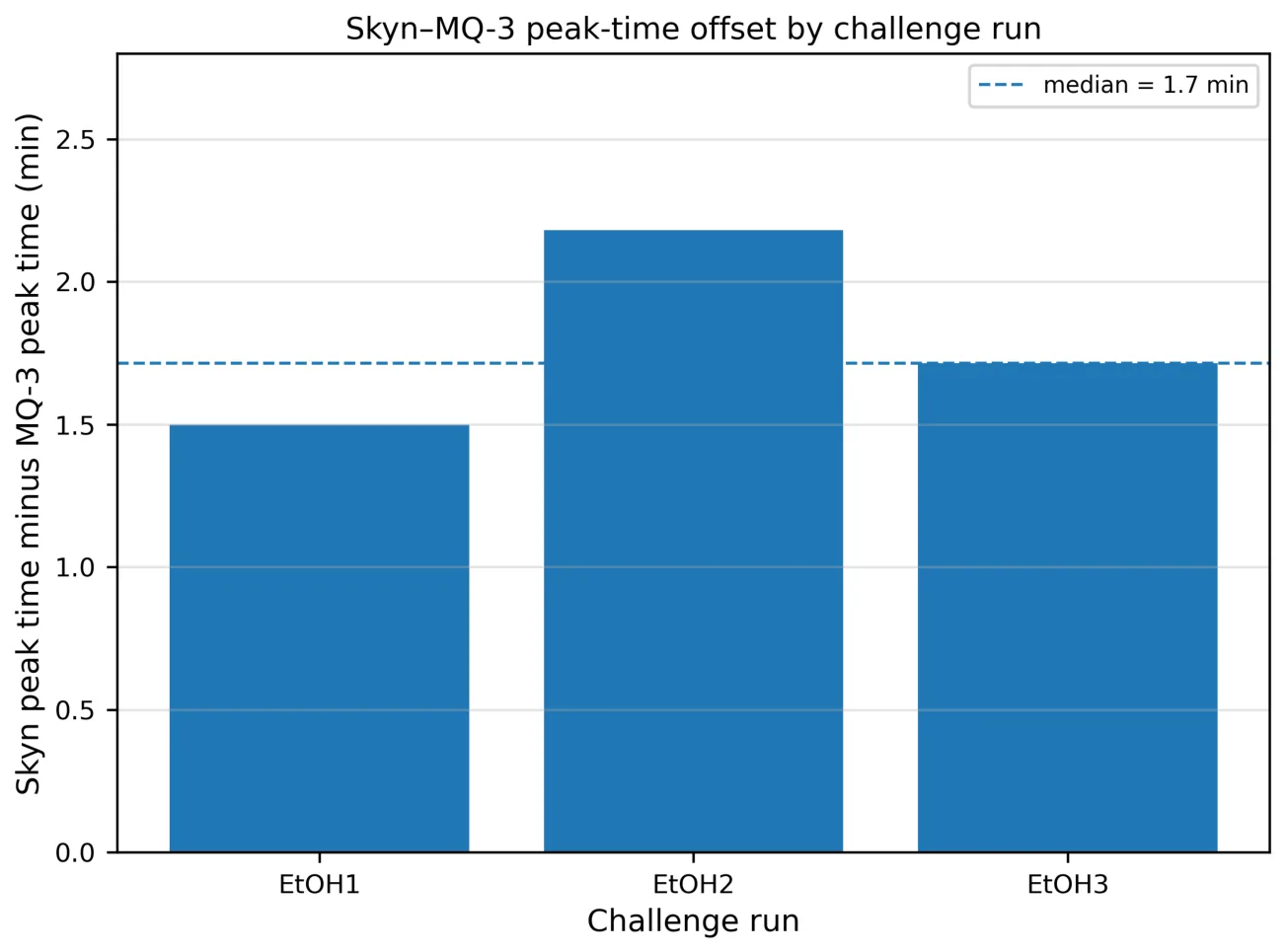
Skyn-minus-MQ-3 peak-time offsets across the three 1 μL denatured-alcohol challenge runs, EtOH1–EtOH3. Bars show run-level peak offsets, and the dashed line shows the median peak offset of 1.716 min. Positive values indicate that the Skyn peak occurred later than the MQ-3 peak. First-difference diagnostics separately supported a shared 2 min alignment shift; alcohol-free controls were not used to estimate the challenge-response alignment offset.

The primary first-difference estimator on the monotonic 1 min analysis time axis identified a 2 min configuration-specific alignment offset between the Skyn and MQ-3 response traces for EtOH1, EtOH2, and EtOH3 within the 15–45 min response-characterization window. The shared alignment shift was, therefore, 2 min across the three challenge runs. Timestamp-based first-difference diagnostics gave 3.0, 2.0, and 1.0 min for EtOH1, EtOH2, and EtOH3, respectively. As a directional-sensitivity check, the first-difference search was repeated over −10 to +10 min; the same +2 min optimum was obtained for EtOH1, EtOH2, and EtOH3. Cumulative-trace diagnostics were retained as supplementary checks because they were not used to define the primary alignment shift. The retrospective leave-one-run-out check selected 2 min in every fold; the held-out Pearson correlations and normalized RMSE values consequently matched the corresponding after-shift values in Table 4. On the $T_{0}$-anchored 20 s grid, the optima were 160, 100, and 80 s for EtOH1–EtOH3, respectively. Moving the bin origin produced ranges of 160–180, 100–140, and 80–100 s (Table S5). The shared 2 min setting was, therefore, specific to the primary 1 min analysis grid. Native-record Skyn-minus-MQ-3 peak-time offsets are shown separately in Figure 3.

### 3.4. Challenge-Run Peak Timing and Shared Alignment

**Figure 4:**
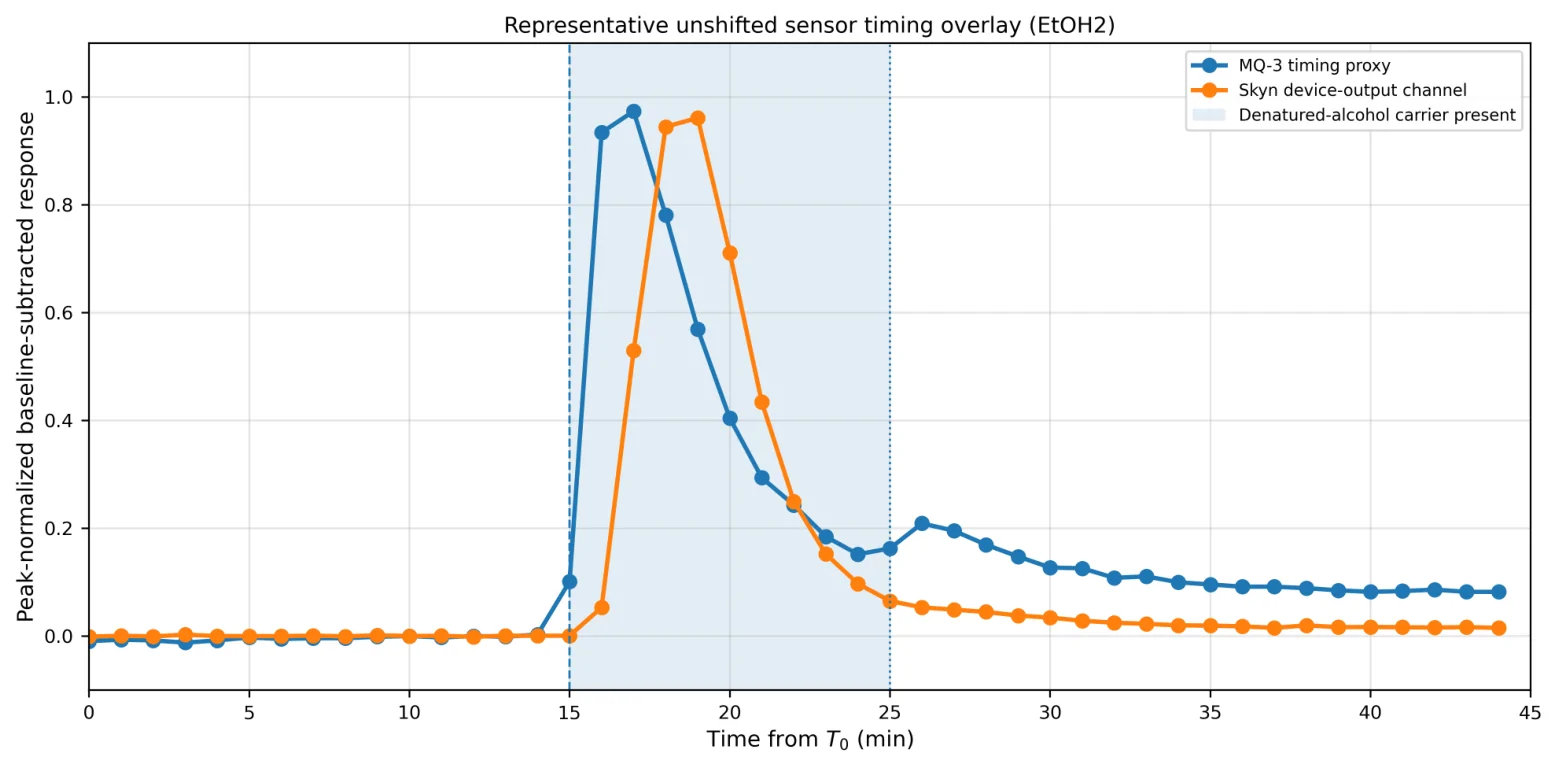
Representative unshifted, local-baseline-corrected and peak-normalized MQ-3/Skyn responses for EtOH2. The alcohol-treated carrier was introduced at 15 min and removed at 25 min. The shaded interval denotes carrier presence, not a constant vapor concentration. The pre-insertion interval and post-removal response are shown; carrier removal did not establish an ethanol-free atmosphere. The plot compares temporal response features and is not a concentration-conversion plot.

Across EtOH1–EtOH3, MQ-3 peak times were 17.334, 17.001, and 17.334 min from $T_{0}$, whereas Skyn peak times were 18.833, 19.183, and 19.050 min. The resulting Skyn-minus-MQ-3 peak offsets were 1.499, 2.183, and 1.716 min, with a median of 1.716 min. MQ-3 peak deltas were 756, 732, and 718 ADC, whereas Skyn peak deltas were 288.348, 106.883, and 102.426 μg L^−1^ export units. Amplitudes were not compared directly across sensor types. Figure 4 shows the unshifted, local-baseline-corrected and peak-normalized MQ-3/Skyn responses for EtOH2.

On the paired 1 min grid, the Skyn-minus-MQ-3 peak-time difference was 2 min before alignment and 0 min after the shared shift in each challenge run. These binned values are distinct from the native-record peak-time offsets of approximately 1.5–2.2 min reported above; coincident peak bins do not establish exact coincidence of the native-record peaks. Decay-shape differences remained after alignment. The shift is, therefore, interpreted as a numerical peak- and response-shape alignment setting, not as a concentration conversion or an estimate of intrinsic sensor latency.

### 3.5. Joint MQ-3/Skyn Response-Window Statistics

**Figure 5:**
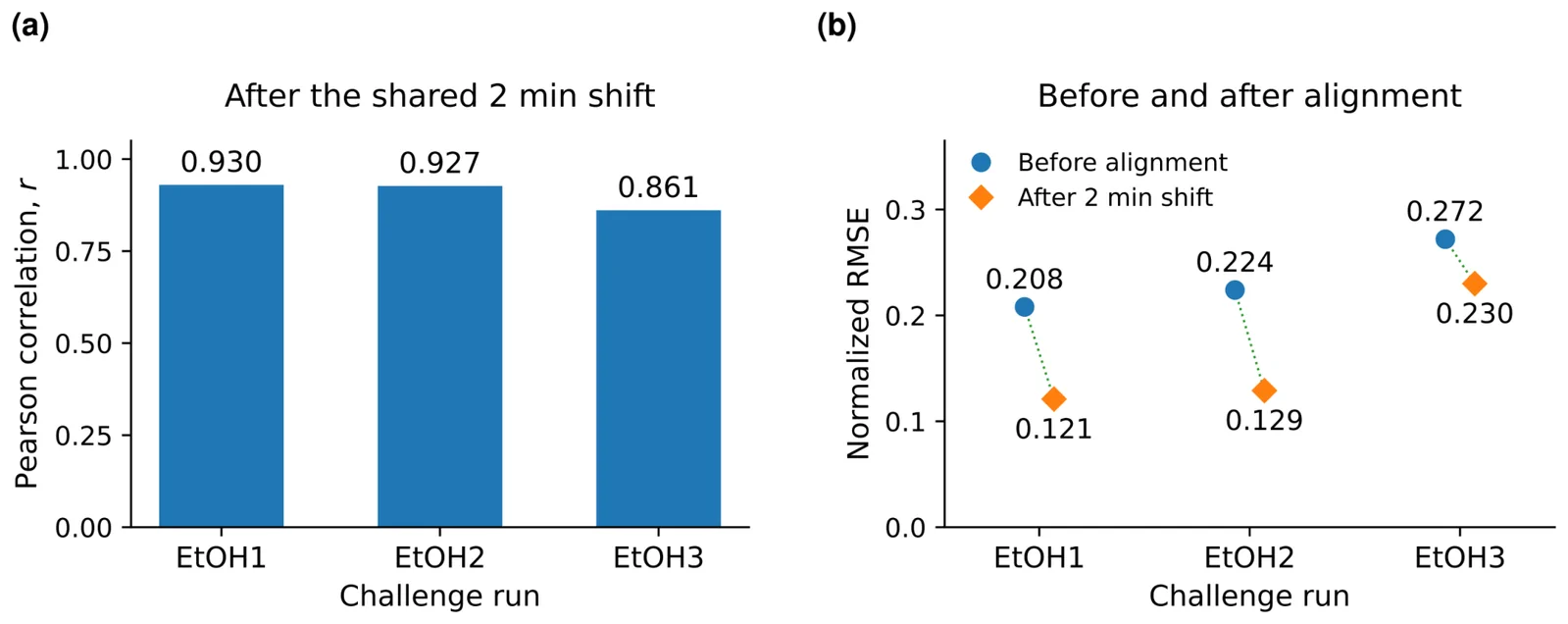
MQ-3/Skyn response-window alignment diagnostics for EtOH1–EtOH3. (**a**) Paired 1 min Pearson correlations after the shared 2 min shift. (**b**) Normalized RMSE before and after the same shift, using the values reported in Table 4. Each run is a technical repetition with the same sensor pair. The metrics describe within-run temporal response similarity, not concentration calibration or independent validation of a transferable offset.

Figure 5 summarizes the after-shift Pearson correlations and the before- and after-shift normalized RMSE values. Pearson correlation increased after the 2 min shift in all three challenge runs, from 0.751 to 0.930 in EtOH1, from 0.686 to 0.927 in EtOH2, and from 0.695 to 0.861 in EtOH3. Normalized RMSE decreased from 0.208 to 0.121, from 0.224 to 0.129, and from 0.272 to 0.230, respectively. Spearman rank correlation decreased in EtOH1 and EtOH2 and increased in EtOH3 (Table 4); the effect of alignment, therefore, depended on the metric. Alcohol-free controls were not used for challenge-response alignment inference. Because the shared shift was selected from these same three challenge traces, the before-and after-shift statistics are interpreted as internal alignment diagnostics; they do not imply raw-unit interchangeability, absolute ethanol calibration, or independent validation of a transferable offset.

### 3.6. Onset-Offset Sensitivity Analysis

Threshold-onset estimates were coincident before shifting and advanced by 1–2 min after the 2 min shift. Accordingly, the shift was interpreted as a peak- and response-shape alignment parameter rather than as a uniform onset-delay correction. Onset results were retained as a supplementary sensitivity diagnostic, whereas peak timing and paired response-window statistics were treated as the principal alignment evidence. Table 3 and Table 4 summarize the response-magnitude, coverage, temporal-alignment, and paired response-window metrics for the analyzed configuration.

## 4. Discussion

### 4.1. Temporal Alignment Under Controlled Headspace Conditions

Under the tested static-headspace configuration, a repurposed BACtrack Skyn and an MQ-3 timing proxy produced temporally related responses during three denatured-alcohol challenges. A shared 2 min shift increased Pearson correlation and reduced normalized RMSE in all three challenge runs, whereas Spearman rank correlation did not improve consistently and threshold-onset timing was advanced after shifting. The 2 min value is, therefore, interpreted as a configuration-specific peak- and response-shape alignment parameter rather than as a uniform sensor delay. Alcohol-free controls remained below challenge-run peak magnitudes, although MQ-3 control traces included negative drift and positive excursions.

### 4.2. Methodological Contribution and Interpretation

The contribution concerns the interpretation of asynchronously recorded outputs from commercially available sensors rather than a new sensing element or alignment algorithm. The combined diagnostics distinguish an improvement in selected response features from evidence for a physical delay. Although all three challenge runs selected a 2 min offset on the primary 1 min grid, the 20 s analysis selected 80–160 s. Agreement on the coarser grid, therefore, did not establish an invariant delay across runs. Similarly, improved Pearson correlation and normalized RMSE did not imply consistent improvement in rank correlation or threshold-onset alignment. These findings support reporting the analysis grid and timing endpoint together with an alignment setting. They do not establish that a minute-scale correction improves tree-status classification or pest-management decisions.

### 4.3. Comparison with Wearable Alcohol Lag Literature

Recent Skyn studies have examined rise rate, peak magnitude, and rise duration in human monitoring [23] and evaluated drinking detection against breath measurements under varied laboratory and field conditions [24]. These studies support attention to temporal features and application-specific validation. They do not establish selectivity, concentration accuracy, or a transferable delay for plant headspace.

Prior studies of wrist-worn alcohol biosensors provide a useful reference point even though the present deployment is not transdermal. In human laboratory settings, wrist-worn Skyn devices have shown shorter lag relative to breath alcohol than ankle-worn SCRAM devices, but the key point is not the absolute value of the delay; it is that the output is delayed and smoothed relative to faster alcohol measures [11]. Practical guidance for Skyn use likewise emphasizes preprocessing, artifact handling, and analytic alignment when interpreting the device as a time-series instrument rather than as an instantaneous alcohol meter [12]. Human transdermal delays include physiological ethanol transport through tissue and skin [11]. That pathway was absent here, but the chamber measurements still do not separate vapor transport, sensor kinetics, device processing, and timestamp effects. However, the approximately 2 min offset observed here should not be compared directly with transdermal human alcohol delays because the Skyn was repurposed in a headspace configuration rather than worn on skin. The estimated offset reflects the complete sensing, timestamping, and preprocessing configuration and is not interpreted as intrinsic sensor latency.

### 4.4. Comparison with Plant VOC Chamber Methodology Literature

The chamber literature emphasizes that material choice, chamber geometry, flow state, and environmental stability are prerequisites for interpretable VOC measurements [14,15]. The current study did not estimate flux or absolute headspace concentration, and the static PLA enclosure should not be interpreted as a dynamic VOC chamber. Instead, it provided repeatable local geometry for comparing timing between two ethanol-responsive channels. This distinction is central: the results support temporal alignment within this configuration, but chamber material, sealing, dead volume, source placement, and air exchange could all alter lag and response shape in future deployments. Because the source was closer to the MQ-3 than to the Skyn (3.1 versus 12.8 cm) in the static chamber, the observed offset may partly reflect vapor transport rather than sensor response alone. No equal-distance or position-swap comparison was performed, so the contributions of chamber transport and sensor/device response could not be separated.

Alcohol-vapor sorption, swelling, and vapor-induced crystallization have been reported for PLA films [25]. These observations do not establish chemical degradation of the printed enclosure under the present conditions. The timing data do not demonstrate long-term material stability or exclude chamber-wall sorption and subsequent release as contributors to response shape.

### 4.5. Role of the MQ-3 Timing Proxy

The Skyn and MQ-3 use different sensing principles: the Skyn is a fuel-cell sensor, whereas the MQ-3 is a heated metal-oxide sensor [11,18]. Because the traces were normalized before comparison, their similar response shapes do not indicate identical hardware. The MQ-3 was used as an uncalibrated timing proxy for within-configuration response comparison; it was not treated as an ethanol-selective analytical reference or concentration standard. All challenge-run peak deltas exceeded the full alcohol-free MQ-3 range, and peak timing was closely clustered across EtOH1–EtOH3. Its directly logged ADC stream and earlier observed peaks provided a comparison channel for this experiment, not a verified measure of vapor arrival or intrinsic response speed. The experiment does not establish that MQ-3 is required in an eventual tree-monitoring system. However, the nonflat MQ-3 behavior in B1–B4 shows that the channel does not independently establish ethanol specificity. Metal-oxide sensor responses are sensitive to temperature, humidity, drift, heater state, sensor aging, and chamber history [16]. The present baseline correction and temporal alignment do not establish chemical selectivity, resistance to drift, or long-term sensor stability. Tests with individual plant volatiles and living plants, as used in recent commercial-sensor work [10], address questions that remain untested by the present mixed-alcohol timing comparison.

### 4.6. Environmental Confounds and Drift Limitations

Environmental variation was modest but not absent across B0–B4 and EtOH1–EtOH3. Temperature and relative humidity were monitored continuously, and environmental summaries were included to identify gross confounding. Challenge-run MQ-3 positive excursions were larger than alcohol-free positive excursions, and the principal peaks occurred after carrier insertion within the scheduled 15–25 min interval, supporting their interpretation as challenge-associated responses under the tested conditions. However, B2 and B4 showed moderate MQ-3 excursions without ethanol addition, and B1 and B3 showed substantial negative MQ-3 drift. These findings show that structured MQ-3 variation can occur without alcohol addition; the present data do not identify its specific cause. The dataset is, therefore, too small to support a reliable environmental compensation model. Both plant VOC measurements and metal-oxide sensing remain sensitive to environmental conditions, and future field interpretation will require stronger environmental control, explicit humidity/temperature compensation, or validation across a broader environmental range [14,15,16].

### 4.7. Limits of Transferability Beyond This Configuration

The study used one sensor pair, one enclosure configuration, one nominal challenge dose, and three technical challenge runs. These runs support a descriptive assessment of timing consistency in this configuration; the 1 min observations within a run are not independent experimental replicates. Identical nominal liquid loading did not produce identical Skyn peak deltas, which ranged from 102.426 to 288.348 μg L^−1^ in export units. The data do not distinguish variation in vapor delivery from sensor variability or exposure-history effects and do not establish complete between-run recovery or absence of carryover. The resulting alignment offset is specific to the tested sensing, acquisition, and preprocessing configuration; its consistency across challenge doses, devices, and deployment conditions remains to be tested.

### 4.8. Relation to Field Deployment Scope

The present results provide a configuration-specific timing basis for subsequent tree-monitoring experiments but do not themselves evaluate tree physiological status or field detection performance. Application beyond the tested laboratory configuration requires separate validation under realistic deployment conditions. Timing was examined as a limited acquisition and processing question, not as a solution to selectivity. Tree monitoring additionally requires tests with ethanol, relevant interfering plant volatiles, and biological samples against an independent analytical reference, as illustrated by recent plant-sensing studies [9,10]. The present experiment does not establish a recommended between-tree recovery period or field sampling duration; these durations should be determined from return-to-baseline tests under field conditions.

## 5. Conclusions

One BACtrack Skyn wearable fuel-cell alcohol sensor and one MQ-3 timing proxy were evaluated in five alcohol-free control runs and three technical 1 μL denatured-alcohol challenge runs. First-difference analysis selected a 2 min alignment setting on the primary 1 min grid in all three challenge runs; the supplementary 20 s analysis gave run-specific optima of 80–160 s. The shared shift increased Pearson correlation and reduced normalized RMSE in every run, whereas rank correlation did not improve consistently. Under the specified retrospective threshold-onset procedure, onset estimates were coincident before shifting and advanced by 1–2 min afterward. The shift, therefore, improved selected peak and response-shape comparisons without providing a uniform threshold-onset correction. Because the carrier center was 3.1 cm from the MQ-3 sensing element and 12.8 cm from the Skyn sensing surface in the static chamber, the recorded timing differences could not be separated into vapor-transport and sensor/device contributions. These results characterize one commercial sensor pair and one chamber configuration; they do not establish an intrinsic sensor latency, a transferable alignment setting, chemical selectivity, concentration calibration, or biological or field detection performance.

For comparable datasets, the processing sequence comprises timestamp and validity checks, definition of a common run origin, signed baseline referencing, median aggregation onto a common grid, first-difference offset estimation, and separate evaluation of peak timing, correlations, normalized shape errors, and threshold-onset sensitivity. Here, Equation (1) defines the startup-baseline-relative MQ-3 response, whereas Equations (2) and (4) define the locally baseline-referenced responses using the 10≤t<15 min analytical interval; the primary response window was 15–45 min on a 1 min grid. Alcohol-free and environmental records provide checks on background variation. Validity rules, analysis windows, grid resolution, and the alignment setting require evaluation for each new configuration; the 2 min setting is not a general correction.

## Figures and Tables

**Table 1:** Experimental configuration and sensor roles for the laboratory setup. The table summarizes the enclosure role, sensing channels, analytical use, sampling interval, and recorded outputs for the controlled headspace experiment.

Component	Role in Study	Detailed Setting	Notes
BACtrack Skyn	Wearable fuel-cell ethanol-responsive channel	One Skyn device mounted in the static headspace	Interpreted as a device-output time series, not a chamber concentration
MQ-3	Uncalibrated timing proxy	One analog MQ-3 channel logged by the ESP32	Used for within-configuration response timing; not used as an ethanol-selective or concentration reference
BME280	Environmental context	Temperature, relative humidity, pressure	Used for confound checks; no correction model fitted
Thermocouple + MAX6675	Local temperature context	One Type-K thermocouple channel	Logged by ESP32 as auxiliary temperature context
ESP32 logger	MQ-3/environment acquisition	20 s logging interval; SD-card CSV output	Logs run_id, epoch_s, ntp_ok, millis_ms, environmental variables, MQ-3 raw ADC, and baseline-referenced MQ-3 response
Headspace enclosure	Standardized exposure geometry	Closed static PLA enclosure; central cuboid chamber 3.3×7.2×14.9 cm; cylindrical channel 30.5 cm length and 2.7 cm internal diameter	Used for repeatable timing comparison, not dynamic flux estimation
Alcohol-free controls	Background and chamber controls	B0–B4; no alcohol added	Analyzed using the same predefined time windows as the challenge runs; used to characterize drift and background excursions
Denatured-alcohol challenge runs	Controlled perturbation runs	EtOH1–EtOH3; technical 1 μL denatured-alcohol challenges	Used to estimate the configuration-specific alignment offset and paired MQ-3/Skyn response metrics

**Table 2:** Preprocessing, quality-control, and alignment parameters used for the analysis. Parameters define the baseline, challenge, response, resampling, quality-control, lag-estimation, merge, and statistical settings used to produce the reported results.

Parameter	Setting Used in This Analysis	Purpose
Dataset	B0–B4 alcohol-free controls plus EtOH1–EtOH3 technical denatured-alcohol challenge runs	Dataset used for reported results
Challenge dose	0 μL for B0–B4; 1 μL denatured alcohol for EtOH1–EtOH3	Alcohol-free controls and controlled challenge runs
Initial quality-control interval	0–15 min	Startup and quality-control context
Analytical baseline	10–15 min	Local pre-exposure baseline correction
Challenge window	15–25 min	Carrier-present interval: treated in EtOH1–EtOH3; untreated in B0–B4
Response-characterization window	15–45 min	Peak timing, paired response statistics, and early decay
Resampling interval	1 min	Common grid for lag and paired statistics
Resampling aggregator	Median	Median aggregation for 1 min resampling and reduced point-level outlier influence
ESP32 logging interval	20 s	Raw MQ-3/environment acquisition
MQ-3 visualization smoothing	5 samples	Visualization only
Skyn TAC cap	500 μg L^−1^ device-export units	Predefined high-value artifact exclusion
Minimum Skyn points	30	Quality-control requirement
Minimum unique Skyn TAC values	10	Quality-control requirement
Primary lag search bounds	0–10 min	Positive values indicate that Skyn trails MQ-3
Directional sensitivity search	−10 to +10 min	Checks whether the directional restriction changes the optimum
Primary lag diagnostic	First-difference lag estimator on the 1 min monotonic time axis	Response-slope alignment diagnostic
Common applied lag	Shared 2 min alignment shift, based on challenge-run first-difference diagnostics	Primary alignment shift for challenge-run display and paired statistics
Merge tolerance	2 min	Exported quality-control files only; not used for the primary paired statistics
Joint response window	15–45 min	Primary paired MQ-3/Skyn response statistics
Circular-shift null	Exact all-valid circular shifts, minimum shift 3 min	Within-run sensitivity diagnostic
Bootstrap	Moving-block bootstrap, block length 5 min, n=1000	Within-run sensitivity interval for paired correlations
Lag estimation window	15–45 min	Response-window lag diagnostics
Minimum lag-overlap points	20	Prevents rejection of short-lag estimates within the 15–45 min window
Forward fill	Not used for primary quantitative analysis	Avoids artificial continuity in lag/correlation diagnostics

**Table 3:** Response-magnitude and coverage summary for five alcohol-free controls and three denatured-alcohol challenge runs. Alcohol-free peak deltas represent maximum positive excursions within the response window or available Skyn response window rather than challenge-response peaks.

Run	Type	MQ-3 Peak Delta, ADC	MQ-3 Minimum Delta, ADC	Skyn Peak Delta, μg L^−1^ Export Units	Skyn Coverage to 45 min	Note
B0	Alcohol-free	72	−7	0.415823	No; ends 43.45 min	Incomplete Skyn coverage
B1	Alcohol-free	63	−193	0.365924	Yes	Negative MQ-3 drift
B2	Alcohol-free	308	−10	0.324342	No; ends 39.55 min	Positive MQ-3 excursion
B3	Alcohol-free	7	−162	0.382557	Yes	Minimal positive MQ-3 response; negative drift
B4	Alcohol-free	240	−41	0.415823	Yes	Broad MQ-3 positive excursion
EtOH1	Challenge	756	−3	288.348285	Yes	1 μL denatured-alcohol challenge
EtOH2	Challenge	732	7	106.883138	Yes	1 μL denatured-alcohol challenge
EtOH3	Challenge	718	−13	102.425516	Yes	1 μL denatured-alcohol challenge

**Table 4:** Challenge-run temporal-alignment and paired response-window statistics before and after applying the shared 2 min alignment shift.

Metric	EtOH1	EtOH2	EtOH3	Summary
MQ-3 peak time, min from $T_{0}$	17.334	17.001	17.334	range = 17.001–17.334
Skyn peak time, min from $T_{0}$	18.833	19.183	19.050	range = 18.833–19.183
Skyn minus MQ-3 peak offset, min	1.499	2.183	1.716	median = 1.716
First-difference alignment offset, min	2.0	2.0	2.0	shared shift = 2 min
Pearson *r*, before shift	0.751	0.686	0.695	range = 0.686–0.751
Pearson *r*, after shift	0.930	0.927	0.861	range = 0.861–0.930
Spearman ρ, before shift	0.894	0.905	0.817	range = 0.817–0.905
Spearman ρ, after shift	0.823	0.890	0.846	range = 0.823–0.890
Normalized RMSE, before shift	0.208	0.224	0.272	range = 0.208–0.272
Normalized RMSE, after shift	0.121	0.129	0.230	range = 0.121–0.230

## Data Availability

The EtOH2 MQ-3 acquisition record and processed paired 1 min MQ-3/Skyn series are provided in Data S1. The remaining data and the analysis code supporting the reported results are available from the corresponding author upon reasonable request, subject to institutional approval and collaborator data-use restrictions. Account- and device-identifying fields are excluded.

## Supplementary Materials

The following supporting information can be downloaded at: https://www.mdpi.com/article/10.3390/s26216949/s1. Figure S1: Response-magnitude separation across alcohol-free controls and denatured-alcohol challenge runs; Figure S2: Paired MQ-3/Skyn response-window correlations before and after the shared 2 min alignment shift; Figure S3: First-difference alignment diagnostic curves; Figure S4: Alcohol-free MQ-3 and Skyn quality-control traces; Figure S5: Per-run normalized MQ-3/Skyn response-shape agreement; Figure S6: Recorded MQ-3 ADC values for EtOH2; Figure S7: Annotated apparatus views and reported source-to-sensor distances; Table S1: Detailed response-window baseline, sample-count, coverage, magnitude, and timing metrics; Table S2: Alignment diagnostics, onset offsets, baseline drift, merge coverage, and offset interpretation; Table S3: Paired MQ-3/Skyn response-window statistics and normalized shape metrics; Table S4: Environmental summaries by run and analysis window; Table S5: Retrospective leave-one-run-out and temporal-grid sensitivity checks; Table S6: Chronology and source-file mapping of the included recordings; Data S1: EtOH2 MQ-3 acquisition record and processed paired 1 min MQ-3/Skyn series.
