# Supplementary material for: Configuration-Specific Temporal Alignment of a Repurposed Wearable Fuel-Cell Alcohol Sensor with an MQ-3 Timing Proxy in a Laboratory Static-Headspace Chamber
Source: Sensors (Basel). 2026 Nov 3;26(21):6949. doi: 10.3390/s26216949 (PMC13766502; doi:10.3390/s26216949)
Supplement: Supplementary file 1 [file sensors-26-06949-s001.zip › sensors_4540771_supplementary.zip!/sensors_4540771_supplementary.pdf]

Supplementary Materials: Configuration-Specific Temporal Alignment of a Repurposed Wearable Fuel-Cell Alcohol Sensor with an MQ-3 Timing Proxy in a Laboratory Static-Headspace Chamber

Hasan Mirzakhaninafchi*, Glen Rains, Wesley Porter, Canicus Mwitta, Denis Kiobia, Shekhar Thapa, Peter Ngimbwa and Christopher Ranger

The following supporting information is included with this article: Figure S1: Response-magnitude separation across alcohol-free controls and denatured-alcohol challenge runs; Figure S2: Paired MQ-3/Skyn response-window correlations before and after the shared 2 min alignment shift; Figure S3: First-difference alignment diagnostic curves; Figure S4: Alcohol-free MQ-3 and Skyn quality-control traces; Figure S5: Per-run normalized MQ-3/Skyn response-shape agreement; Figure S6: Recorded MQ-3 ADC values for EtOH2; Figure S7: Annotated apparatus views and reported source-to-sensor distances; Table S1: Detailed response-window baseline, sample-count, coverage, magnitude, and timing metrics; Table S2: Alignment diagnostics, onset offsets, baseline drift, merge coverage, and offset interpretation; Table S3: Paired MQ-3/Skyn response-window statistics and normalized shape metrics; Table S4: Environmental summaries by run and analysis window; Table S5: Retrospective leave-one-run-out and temporal-grid sensitivity checks; Table S6: Chronology and source-file mapping of the included recordings; Data S1: EtOH2 MQ-3 acquisition record and processed paired 1 min MQ-3/Skyn series.

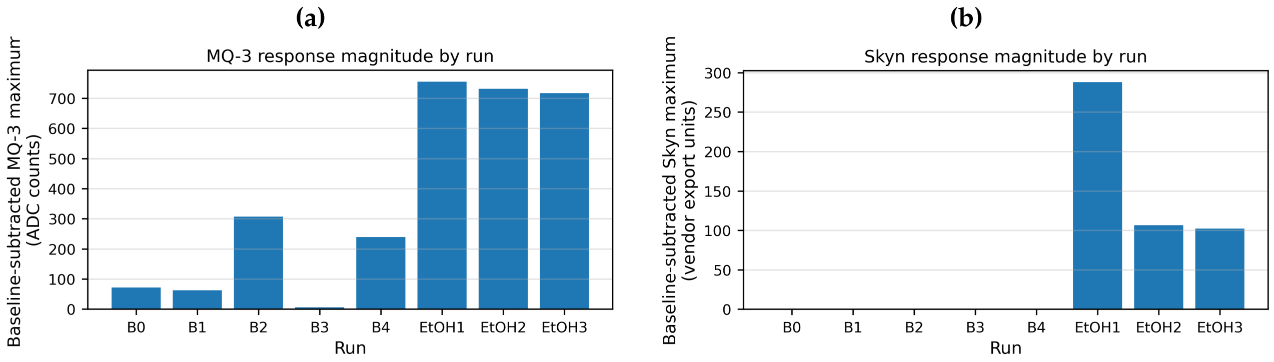

Figure S1. Response-magnitude separation across alcohol-free controls and denatured-alcohol challenge runs. (a) MQ-3 local-baseline-corrected positive peak delta by run. (b) Skyn local-baseline-corrected positive peak delta by run. Alcohol-free controls remained below challenge-run peak-delta ranges for both channels, although MQ-3 alcohol-free traces showed nonflat drift or positive excursions in some runs.

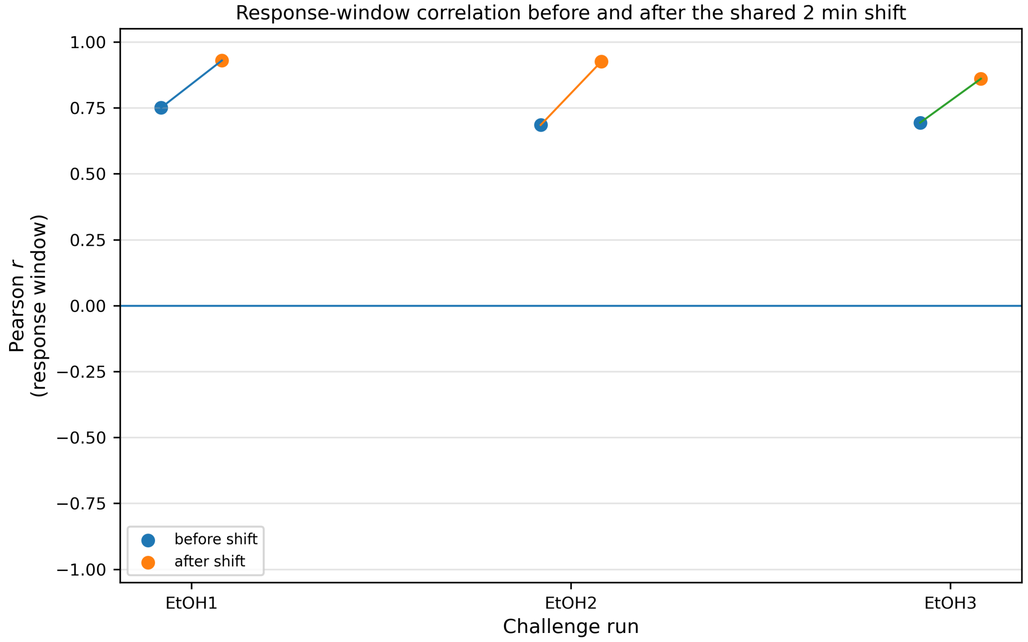

Figure S2. Paired MQ-3/Skyn response-window correlations before and after applying the shared 2 min alignment shift to EtOH1–EtOH3. The figure shows the effect of the shared alignment shift on cross-sensor time-course association in the 15–45 min response-characterization window.

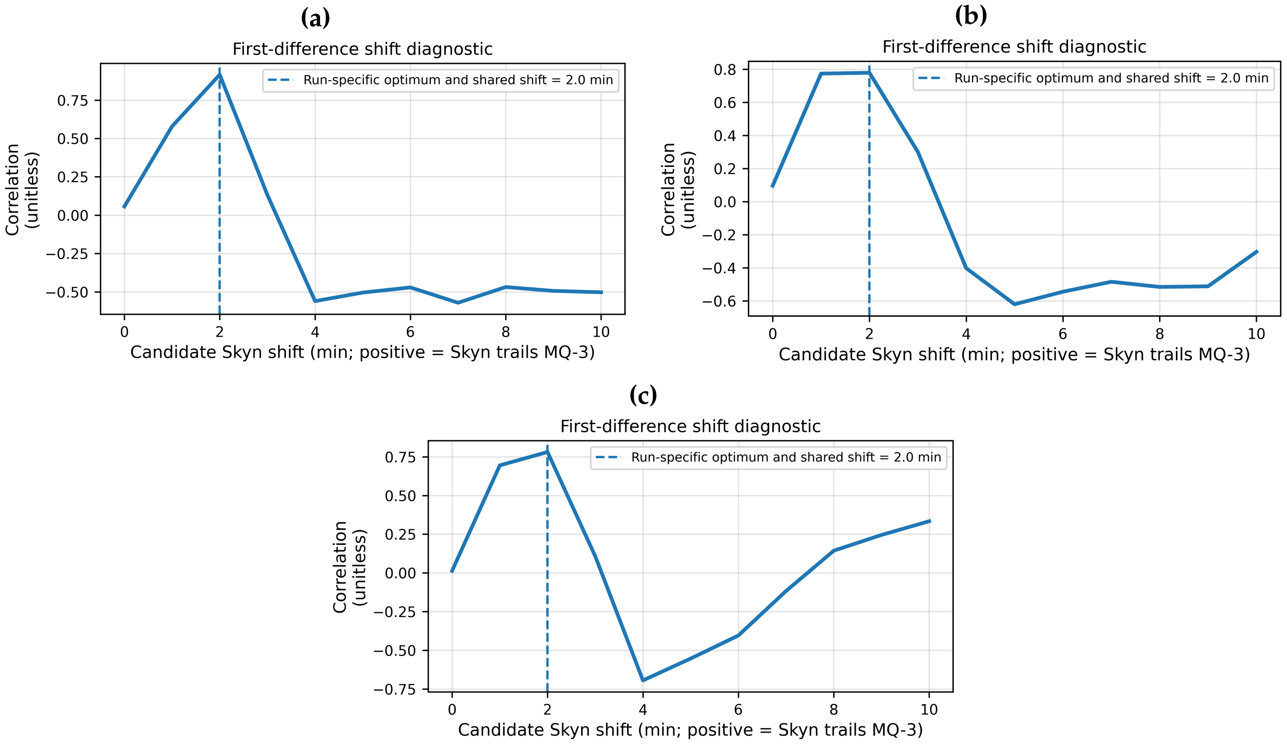

Figure S3. First-difference alignment diagnostic curves for EtOH1–EtOH3 on the monotonic 1 min analysis time axis. The plotted primary search used the 15–45 min response-characterization window and a 0–10 min search range. A separate directional-sensitivity search over −10 to +10 min produced the same +2 min optimum for all three challenge runs.

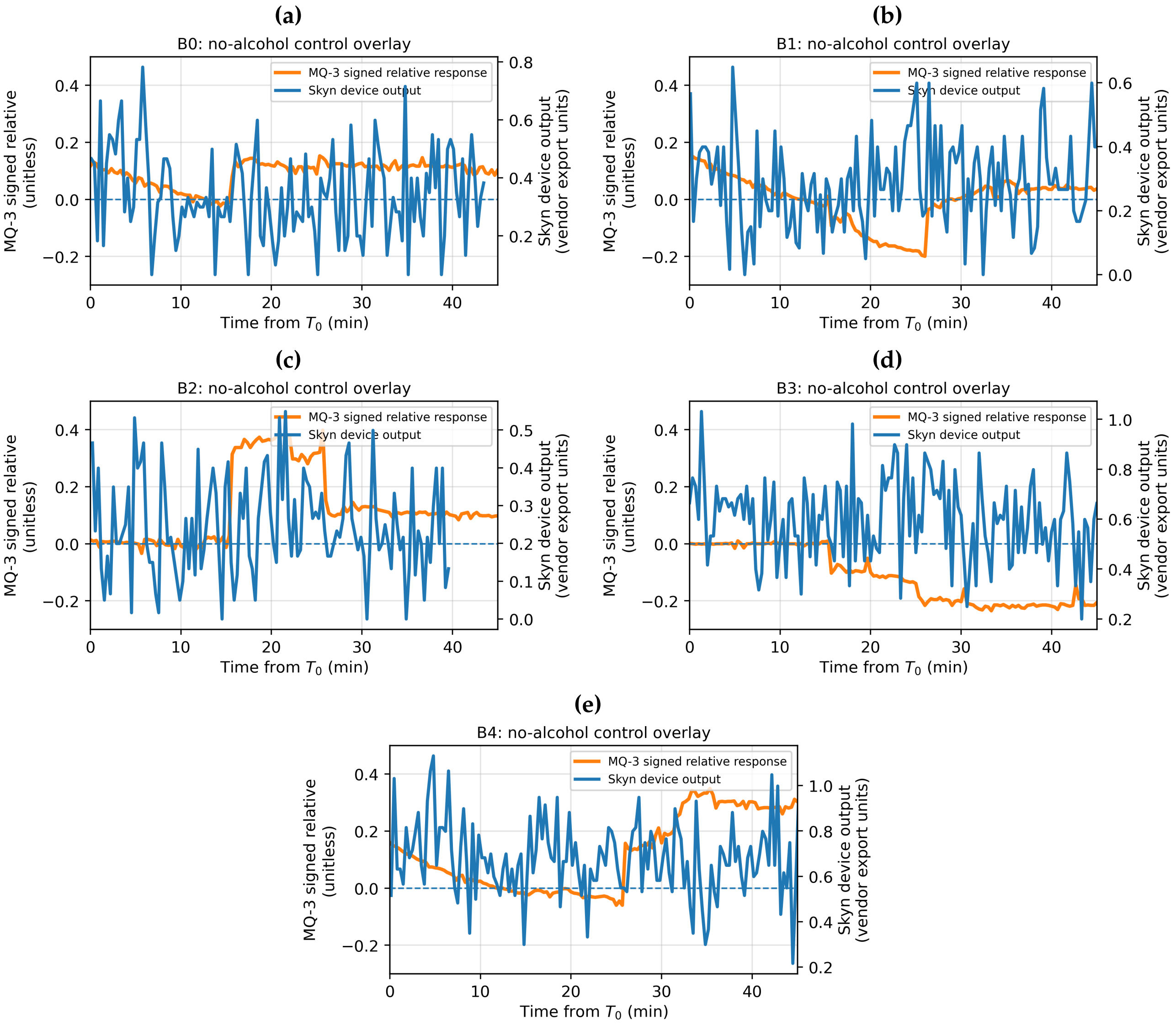

Figure S4. Alcohol-free MQ-3 and Skyn response-shape quality-control traces for B0–B4. These traces show that alcohol-free Skyn responses remained at blank scale, while MQ-3 alcohol-free traces included negative drift and positive excursions. Alcohol-free runs were used as background controls and were not used for ethanol-response lag estimation.

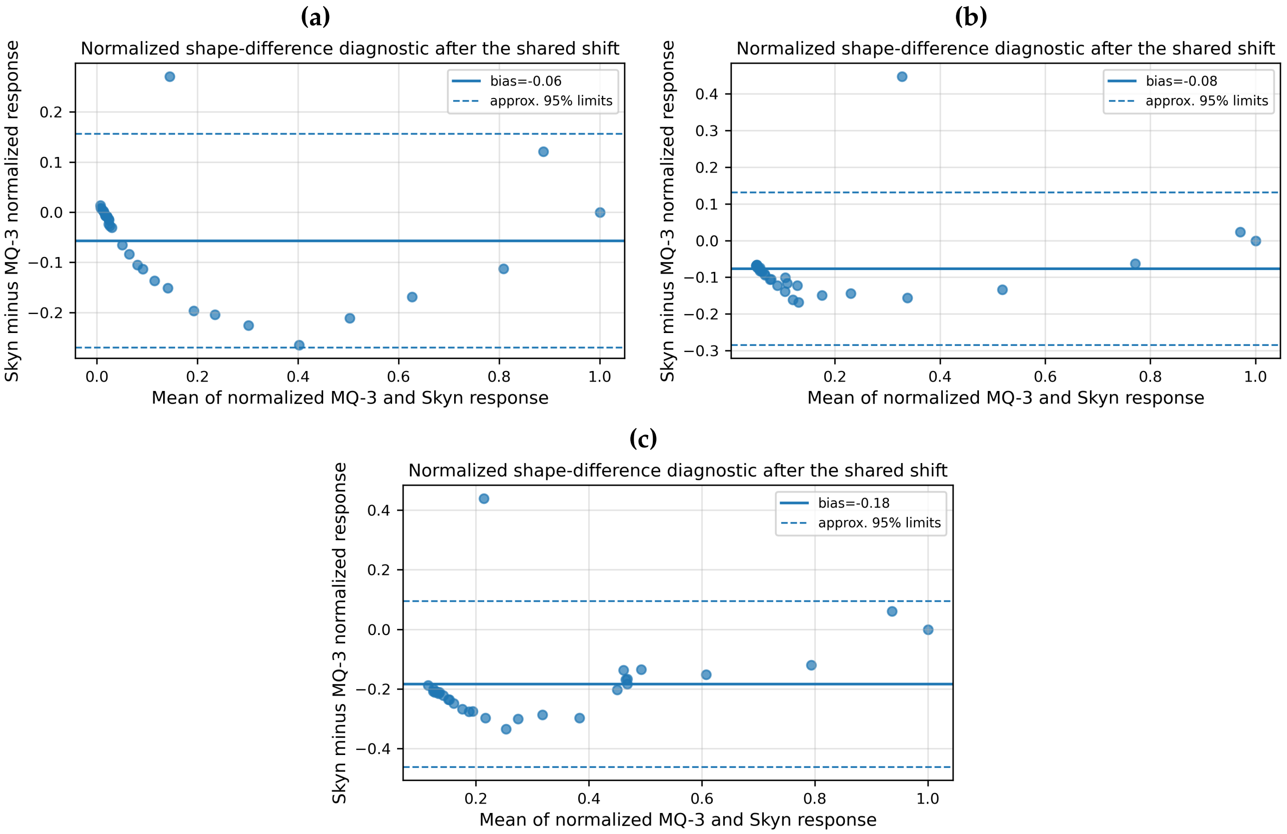

Figure S5. Per-run normalized MQ-3/Skyn response-shape agreement after applying the shared 2 min alignment shift: (a) EtOH1, (b) EtOH2, and (c) EtOH3. Normalized bias is the mean paired difference between peak-normalized responses, calculated as Skyn minus MQ-3. The post-alignment normalized bias for EtOH1 is −0.057.

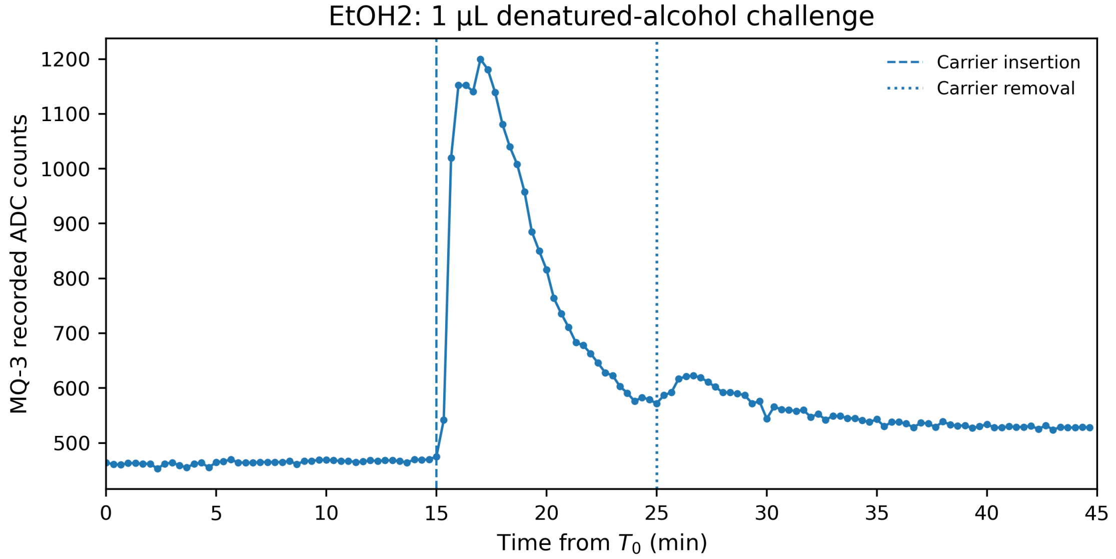

Figure S6. Recorded MQ-3 ADC values for EtOH2 during a 1 μL denatured-alcohol challenge. Points retain the original sampling times and ADC values within the 0–45 min interval; no baseline subtraction, normalization, smoothing, resampling, or temporal alignment shift was applied. Vertical lines mark carrier insertion at 15 min and removal at 25 min. The post-removal interval is not a verified zero-ethanol exposure. The recorded MQ-3 data and the processed paired 1 min MQ-3/Skyn series are provided separately in Data S1.

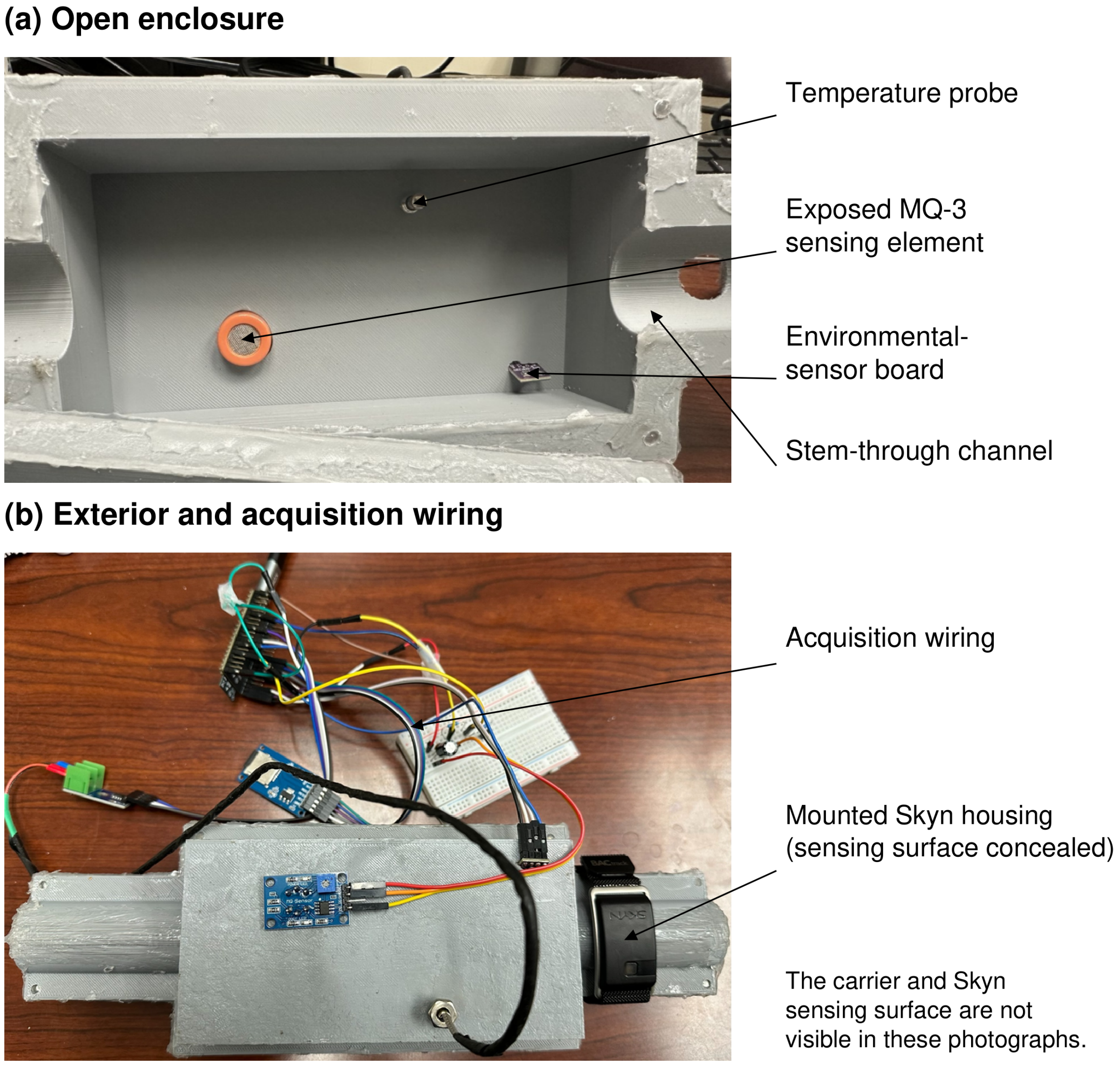

Figure S7. Annotated apparatus views and reported source-to-sensor distances. (a) Open enclosure with annotations identifying the exposed MQ-3 sensing element, environmental-sensor board, temperature probe, and stem-through channel. (b) Exterior view identifying the mounted Skyn housing and acquisition wiring. The filter-paper carrier and Skyn sensing surface are not visible in these photographs. The measured straight-line distances from the carrier center were 3.1 cm to the MQ-3 sensing element and 12.8 cm to the Skyn sensing surface, as reported in Section 2.4 of the main article. These distances were measured separately and were not inferred from the photographs.

Table S1. Detailed response-window baseline, sample-count, and Skyn coverage metrics for B0–B4 alcohol-free controls and EtOH1–EtOH3 denatured-alcohol challenge runs.

Run	Type	Dose (μL)	MQ-3 baseline ADC median	Skyn baseline (μg L^{-1} export units) median	MQ-3 response n	Skyn response n	Skyn observed response window (min)	Full Skyn 15–45 min coverage
B0	Alcohol-free	0.0	410	0.299	90	85	15.10–43.45	No
B1	Alcohol-free	0.0	814	0.233	90	90	15.10–44.77	Yes
B2	Alcohol-free	0.0	762	0.225	90	72	15.22–39.55	No
B3	Alcohol-free	0.0	688	0.599	90	90	15.33–45.00	Yes
B4	Alcohol-free	0.0	576	0.632	90	90	15.15–44.82	Yes
EtOH1	Challenge	1.0	467	0.699	90	90	15.17–44.83	Yes
EtOH2	Challenge	1.0	468	0.416	90	90	15.18–44.85	Yes
EtOH3	Challenge	1.0	367	0.566	90	90	15.05–44.72	Yes

Table S1 continued. Response magnitude and timing metrics for B0–B4 alcohol-free controls and EtOH1–EtOH3 denatured-alcohol challenge runs.

Run	MQ-3 peak delta (ADC)	MQ-3 minimum delta (ADC)	MQ-3 peak time from T_0 (min)	Skyn peak delta (μg L^{-1} export units)	Skyn minimum delta (μg L^{-1} export units)	Skyn peak time from T_0 (min)	Skyn–MQ-3 peak offset (min)	QC note
B0	72	-7	25.335	0.416	-0.233	34.783	9.449	Original alcohol-free; incomplete Skyn response-window coverage
B1	63	-193	35.001	0.366	-0.233	25.100	-9.901	Negative MQ-3 drift; full Skyn coverage
B2	308	-10	25.667	0.324	-0.225	21.550	-4.117	Positive MQ-3 excursion; incomplete Skyn coverage
B3	7	-162	15.334	0.383	-0.399	18.000	2.666	Minimal positive MQ-3 response with negative drift; full Skyn coverage
B4	240	-41	35.335	0.416	-0.416	42.167	6.831	Broad MQ-3 positive excursion without ethanol-scale Skyn response; full Skyn coverage
EtOH1	756	-3	17.334	288.348	-0.100	18.833	1.499	Accepted 1 μL denatured-alcohol challenge run; full Skyn coverage
EtOH2	732	7	17.001	106.883	-0.017	19.183	2.183	Accepted 1 μL denatured-alcohol challenge run; full Skyn coverage
EtOH3	718	-13	17.334	102.426	-0.233	19.050	1.716	Accepted 1 μL denatured-alcohol challenge run; full Skyn coverage

Table S2. Lag diagnostics and lag-use status for B0–B4 and EtOH1–EtOH3.

Run	Type	Used for shared alignment shift	Applied shift (min)	Primary-difference offset, monotonic time (min)	Primary-difference correlation, monotonic time	Cumulative offset, monotonic time (min)	Timestamp-difference offset (min)	Timestamp-cumulative offset (min)
B0	Alcohol-free	No	–	2.0	0.313	8.0	0.0	9.0
B1	Alcohol-free	No	–	1.0	0.315	0.0	4.0	0.0
B2	Alcohol-free	No	–	3.0	0.517	2.0	3.0	0.0
B3	Alcohol-free	No	–	7.0	0.372	–	4.0	–
B4	Alcohol-free	No	–	10.0	0.227	0.0	8.0	0.0
EtOH1	Denatured alcohol	Yes	2.0	2.0	0.916	0.0	3.0	1.0
EtOH2	Denatured alcohol	Yes	2.0	2.0	0.779	6.0	2.0	7.0
EtOH3	Denatured alcohol	Yes	2.0	2.0	0.781	0.0	1.0	0.0

Table S2 continued. Peak/onset offsets, baseline drift, merge coverage, and lag interpretation for B0–B4 and EtOH1–EtOH3.

Run	Raw Skyn–MQ-3 peak offset (min)	Unshifted threshold-onset offset, Skyn–MQ-3 (min)	MQ-3 baseline slope (min^{-1})	Skyn merge coverage	Lag interpretation
B0	9.449	1.0	-0.00804	0.979	Alcohol-free QC only; not used for ethanol-response lag estimation
B1	-9.901	–	-0.00810	1.000	Alcohol-free QC only; not used for ethanol-response lag estimation
B2	-4.117	–	0.00776	0.896	Alcohol-free QC only; not used for ethanol-response lag estimation
B3	2.666	–	0.00029	1.000	Alcohol-free QC only; not used for ethanol-response lag estimation
B4	6.831	–	-0.00825	1.000	Alcohol-free QC only; not used for ethanol-response lag estimation
EtOH1	1.499	0.0	-0.00186	0.992	Denatured-alcohol challenge run used for shared-alignment estimation
EtOH2	2.183	0.0	0.00060	1.000	Denatured-alcohol challenge run used for shared-alignment estimation
EtOH3	1.716	0.0	-0.00471	1.000	Denatured-alcohol challenge run used for shared-alignment estimation

Table S3. Paired MQ-3/Skyn response-window correlation statistics before and after shared 2 min alignment shift. Bootstrap intervals and circular-shift statistics are within-run sensitivity diagnostics, not tests of improvement after alignment or independent validation of the selected shift.

Run	Alignment	Applied lag (min)	Paired 1 min observations (n)	Pearson r	Pearson block-bootstrap CI	Spearman ρ	Spearman block-bootstrap CI	Circular-shift p	Circular-shift n
EtOH1	Before alignment shift	0.0	31	0.751	0.639 to 0.981	0.894	0.723 to 0.986	0.037	26
EtOH1	After shared 2 min alignment shift	2.0	31	0.930	0.872 to 0.977	0.823	0.578 to 0.992	0.037	26
EtOH2	Before alignment shift	0.0	31	0.686	0.557 to 0.981	0.905	0.805 to 0.967	0.037	26
EtOH2	After shared 2 min alignment shift	2.0	31	0.927	0.826 to 0.993	0.890	0.739 to 0.972	0.037	26
EtOH3	Before alignment shift	0.0	31	0.695	0.520 to 0.981	0.817	0.527 to 0.990	0.037	26
EtOH3	After shared 2 min alignment shift	2.0	31	0.861	0.768 to 0.993	0.846	0.647 to 0.994	0.037	26

Table S3 continued. Normalized shape and peak-timing statistics before and after the shared 2 min alignment shift. Normalized bias is the mean paired difference between peak-normalized responses, calculated as Skyn minus MQ-3.

Run	Alignment	Maximum null $\|r\|$	Normalized bias, Skyn–MQ-3	Normalized shape RMSE	Normalized shape MAE	MQ-3 peak time in window (min)	Skyn peak time in window (min)	Skyn–MQ-3 peak-time difference (min)
EtOH1	Before alignment shift	0.665	−0.057	0.208	0.093	17.0	19.0	2.0
EtOH1	After shared 2 min alignment shift	0.561	−0.057	0.121	0.084	17.0	17.0	0.0
EtOH2	Before alignment shift	0.659	−0.076	0.224	0.145	17.0	19.0	2.0
EtOH2	After shared 2 min alignment shift	0.389	−0.076	0.129	0.107	17.0	17.0	0.0
EtOH3	Before alignment shift	0.546	−0.184	0.272	0.224	17.0	19.0	2.0
EtOH3	After shared 2 min alignment shift	0.478	−0.183	0.230	0.216	17.0	17.0	0.0

Table S4. Temperature, relative humidity, pressure, and thermocouple summaries by run and analysis window.

Run	Type	Window	Rows (n)	BME280 temp. mean (range), °C	RH mean (range), %	Pressure mean (range), hPa	Thermocouple temp. mean (range), °C
B0	Alcohol-free	Analytical baseline, 10–15 min	15	25.26 (25.22–25.30)	53.74 (53.67–53.86)	1006.55 (1006.52–1006.62)	25.82 (25.50–26.25)
B0	Alcohol-free	Challenge, 15–25 min	30	25.24 (25.21–25.26)	54.40 (53.71–55.78)	1006.47 (1006.39–1006.54)	25.68 (25.00–26.00)
B0	Alcohol-free	Response characterization, 15–45 min	90	25.23 (25.18–25.26)	54.29 (53.71–55.78)	1006.36 (1006.14–1006.54)	25.59 (24.75–26.00)
B1	Alcohol-free	Analytical baseline, 10–15 min	15	24.82 (24.81–24.83)	56.48 (56.34–56.58)	1005.74 (1005.71–1005.79)	25.35 (25.00–25.75)
B1	Alcohol-free	Challenge, 15–25 min	30	24.90 (24.82–24.93)	56.61 (56.19–57.09)	1005.75 (1005.66–1005.85)	25.34 (24.75–25.75)
B1	Alcohol-free	Response characterization, 15–45 min	90	24.78 (24.57–24.93)	57.80 (56.19–59.50)	1005.87 (1005.66–1006.03)	25.24 (24.50–25.75)
B2	Alcohol-free	Analytical baseline, 10–15 min	15	24.74 (24.65–24.81)	56.20 (55.46–56.69)	1005.71 (1005.67–1005.75)	25.08 (24.75–25.50)
B2	Alcohol-free	Challenge, 15–25 min	30	24.62 (24.55–24.73)	57.66 (56.72–59.01)	1005.59 (1005.54–1005.66)	25.02 (24.25–25.50)
B2	Alcohol-free	Response characterization, 15–45 min	90	24.77 (24.55–24.88)	56.65 (55.23–59.01)	1005.61 (1005.54–1005.73)	25.00 (24.25–25.50)
B3	Alcohol-free	Analytical baseline, 10–15 min	15	24.54 (24.40–24.68)	55.91 (55.72–56.01)	1013.08 (1013.05–1013.11)	24.90 (24.25–25.25)
B3	Alcohol-free	Challenge, 15–25 min	30	24.41 (24.29–24.57)	56.58 (55.79–57.22)	1013.09 (1013.03–1013.13)	24.77 (24.25–25.25)
B3	Alcohol-free	Response characterization, 15–45 min	90	24.61 (24.29–24.81)	54.55 (52.80–57.22)	1013.11 (1013.03–1013.21)	24.96 (24.25–25.50)
B4	Alcohol-free	Analytical baseline, 10–15 min	15	24.56 (24.51–24.62)	59.21 (59.14–59.32)	1012.45 (1012.36–1012.54)	24.87 (24.50–25.25)
B4	Alcohol-free	Challenge, 15–25 min	30	24.69 (24.63–24.72)	59.06 (58.73–59.27)	1012.54 (1012.48–1012.62)	25.01 (24.50–25.50)
B4	Alcohol-free	Response characterization, 15–45 min	90	24.74 (24.63–24.81)	58.11 (55.38–59.27)	1012.70 (1012.48–1012.85)	25.04 (24.50–25.50)
EtOH1	Challenge	Analytical baseline, 10–15 min	15	24.44 (24.40–24.47)	60.64 (60.49–60.81)	1005.43 (1005.39–1005.48)	25.03 (24.75–25.50)
EtOH1	Challenge	Challenge, 15–25 min	30	24.43 (24.36–24.49)	60.07 (59.66–60.48)	1005.48 (1005.41–1005.53)	25.02 (24.75–25.25)
EtOH1	Challenge	Response characterization, 15–45 min	90	24.43 (24.26–24.67)	59.77 (59.02–60.48)	1005.40 (1005.30–1005.53)	24.96 (24.50–25.50)
EtOH2	Challenge	Analytical baseline, 10–15 min	15	24.84 (24.79–24.91)	57.55 (57.11–58.14)	1003.17 (1003.13–1003.20)	25.35 (25.00–26.00)
EtOH2	Challenge	Challenge, 15–25 min	30	24.91 (24.82–24.98)	57.40 (57.26–57.60)	1003.11 (1003.05–1003.16)	25.44 (25.00–25.75)
EtOH2	Challenge	Response characterization, 15–45 min	90	24.84 (24.66–24.98)	57.30 (55.45–58.15)	1003.09 (1003.00–1003.16)	25.27 (24.50–25.75)
EtOH3	Challenge	Analytical baseline, 10–15 min	15	24.27 (24.15–24.36)	59.00 (58.63–59.14)	1003.25 (1003.19–1003.32)	24.65 (24.25–25.00)
EtOH3	Challenge	Challenge, 15–25 min	30	24.28 (24.13–24.46)	59.37 (58.94–59.75)	1003.10 (1003.02–1003.20)	24.68 (24.00–25.25)
EtOH3	Challenge	Response characterization, 15–45 min	90	24.38 (24.13–24.62)	59.37 (58.94–59.75)	1002.91 (1002.68–1003.20)	24.77 (24.00–25.25)

Table S5. Retrospective leave-one-run-out and temporal-grid sensitivity checks. Each held-out run used the median of the other two runs' primary 1 min-grid optima. Correlation and error entries show held-out values before/after this shift, using 31 paired 1 min observations. The 20 s optima used 83, 86, and 87 finite paired first differences in EtOH1–EtOH3. Bin-origin ranges cover origin shifts of 0–19 s in 1 s steps and are numerical sensitivity ranges, not confidence intervals.

Run	1 min optimum (s)	Training-pair shift (s)	20 s optimum (s)	Bin-origin range (s)	Pearson r, before/after	Normalized RMSE, before/after
EtOH1	120	120	160	160–180	0.751/0.930	0.208/0.121
EtOH2	120	120	100	100–140	0.686/0.927	0.224/0.129
EtOH3	120	120	80	80–100	0.695/0.861	0.272/0.230

Table S6. Chronology and source-file mapping of the included MQ-3 recordings. Order is derived from the recorded epoch timestamps; canonical labels do not encode collection order. Local times use America/New_York (UTC−04:00). Start/end denote first/last retained samples on the listed date, not power-on/off or carrier insertion/removal. Gaps are measured from the preceding included file's end; they do not establish washout or exclude intervening experiments. B0–B4 are the five analyzed control labels.

Order	Run	MQ-3 source file	Date	Start	End	Gap (h:m:s)
1	B0	`E0_blank.csv`	2026-05-27	14:25:25	15:11:05	–
2	EtOH1	`E1_eth_1uL.csv`	2026-05-28	09:47:41	11:55:41	18:36:36
3	EtOH2	`E2_eth_1uL.csv`	2026-05-28	15:09:20	17:09:21	03:13:39
4	EtOH3	`E3_eth_1uL.csv`	2026-05-29	14:16:47	16:29:47	21:07:26
5	B1	`E02_blank.csv`	2026-06-02	15:30:19	16:16:39	95:00:32
6	B2	`E03_blank.csv`	2026-06-02	16:36:32	17:23:32	00:19:53
7	B4	`E3_blank.csv`	2026-06-03	09:07:35	09:54:15	15:44:03
8	B3	`E1_blank.csv`	2026-06-03	10:11:05	10:59:45	00:16:50
